# Scholarly Publications and Opinions Through 366-Day War on Gaza (2023-2024): A Scoping Review and Bibliometric Analysis

**DOI:** 10.34172/ijhpm.8809

**Published:** 2025-04-14

**Authors:** Emna Ennouri, Mohamed Boussarsar, Chourouk Ben Mahfoudh, Khamis Elessi, Helmi Ben Saad

**Affiliations:** ^1^Faculty of Medicine of Sousse, University of Sousse, Sousse, Tunisia.; ^2^Medical Intensive Care Unit, Farhat Hached University Hospital, Sousse, Tunisia.; ^3^Research Laboratory “Heart Failure”, Farhat Hached University Hospital, Sousse, Tunisia.; ^4^Faculty of Medicine of Tunis, University of Tunis El Manar, Tunis, Tunisia.; ^5^Evidence-Based Medicine Unit, Faculty of Medicine - Islamic University of Gaza, Gaza, Palestine.; ^6^Laboratory of Physiology and Functional Explorations, Farhat Hached University Hospital, Sousse, Tunisia.

**Keywords:** Scholarly Publishing, Gaza Strip (Palestine), Genocide, Israel, Lexical Fields, Decolonizing

## Abstract

**Background:**

The 2023-2024 Gaza Genocide has generated notable scholarly discourse, influenced by various historical, political, and social contexts. These academic writings, rooted in the longstanding "war of words," illustrate how language serves as a potent weapon in conflicts. The present study aimed to analyze the academic response to the 2023-2024 War on Gaza, focusing on the different perspectives, opinions, and lexical choices in scholarly articles.

**Methods:**

A scoping review and bibliometric analysis were conducted on articles from PubMed, pertaining to the 2023-2024 War on Gaza, spanning from October 7, 2023, to October 7, 2024. PRISMA-ScR (Preferred Reporting Items for Systematic Reviews and Meta-Analyses extension for Scoping Reviews) guidelines were used. Individual relevant papers’ data were systematically extracted using a pre-tested form. Articles were categorized based on their stances as pro-Gaza, pro-Israel, or Neutral. Statistical analyses compared the bibliometric data of pro-Gaza and pro-Israel papers, identifying significant associated lexical fields. Factors explaining the different stances were uncovered.

**Results:**

Out of 640 articles identified, 221 were included in the review. Among these, 126 (57%), pro-Gaza, 70 (31.7%), pro-Israel, and 25 (11.3%), Neutral. Pro-Gaza papers, often published in high-ranked journals with global affiliations, focused on humanitarian issues, called for a ceasefire and decried the genocide. Conversely, pro-Israel papers, often from local journals and affiliated with Israeli institutions, focused on political and psychosocial aspects, emphasizing self-defense narratives. Terms independently associated with pro-Gaza positions included "Gaza" in the title, "occupation," "genocide," "punishment," and "ceasefire." Pro-Israel papers featured "Israel" in the title, references to "October 7," and mentions of "Hamas."

**Conclusion:**

This study highlights that academic narratives are profoundly influenced by historical contexts, media portrayal, official discourses, and the authors’ socio-political environments. These findings underscore the intricate connection between scholarly discourse and the broader context of chronic occupation, revealing significant limitations in current global health strategies and highlighting the need to integrate humanitarian crises into these frameworks.

## Background

 The tragic situation in Gaza, where over 42 000 Palestinians have been killed by The Israeli army and more than 1.9 million repeatedly displaced^1^ since the onset of one of the most destructive bombing campaigns,^2^ represents one of the darkest chapters in contemporary history. Several reports from United Nations (UN) agencies have already warned, since the beginning, of the genocide unfolding among a population besieged since 2007.^3,4^ Within the context of decades-long occupation, Gaza, which is a densely populated territory, often labeled as “the world’s largest open-air prison,”^5^ has witnessed the worst form of human suffering. Beyond the extensive human lives losses inflicted,^6^ the offensive on Gaza has also led to widespread destruction of infrastructure,^7^ monumental humanitarian challenges, including starvation,^8^ and a devastating health crisis.^9^ Even efforts to provide humanitarian aid are hindered by the Israeli Army^8,10^ and the deliberate destruction of vital infrastructure such as hospitals.^11^

 Faced with this characterized one-year escalating genocide and in a context where international responses have been slow to materialize, scholars from around the world have mobilized fervently.^12,13^ Probably driven by a deep sense of human responsibility, they have taken a stand, published research, and engaged in debates to raise awareness about the situation in Gaza.^14-19^ In their approaches, different lexical fields are mobilized to support their divergent opinions.^20^ These lexical fields find their roots in the historical constructs of each side,^21^ having culminated over many years into a real “war of words,”^22-24^ a subject of various historiographical studies that demonstrate their characteristics as a true weapon of war.^25^ The ongoing “war of words” surrounding the Israeli-Palestinian conflict is deeply rooted in competing historical narratives, shaping political discourse and public perception.^26^ This struggle over legitimacy dates back to the late 19th century with the emergence of Zionist movements and the establishment of settlements on Palestinian land, culminating in the 1948 Nakba—the forced displacement of Palestinians and destruction of their communities. Subsequent events, including the occupation of the West Bank and Gaza in 1967, the expansion of Israeli settlements, and the blockade of Gaza, have perpetuated the injustice.^27^ While Palestinian resistance is framed as a fight for self-determination and basic rights, Israeli policies, including military offensives and settlement expansions, continue under the pretext of self-defense. These historical and political dynamics shape the polarized narratives that influence academic and public discourse today.^28^ Words can serve as a potent tool, where every term is imbued with a specific connotation that can consciously or unconsciously evoke a particular emotion or reaction in the recipient.^29^ Thus, the strategic use of language can shape perceptions, sway opinions, and reinforce ideological narratives in powerful ways.^26,30^

 Through examining scientific publications, we sought to understand how the academic community contributes to influencing the debate on this major humanitarian crisis.^31^ This scoping review and bibliometric analysis aimed to explore the various perspectives, opinions, and lexical fields expressed by scholars, and their trends while providing an in-depth analysis of the historical and geopolitical context surrounding the War on Gaza.

## Methods

###  Study Design

 This is a scoping review and bibliometric analysis of academic papers whose contents pertain to the ongoing 2023-2024 War on Gaza from October 7, 2023, to October 7, 2024. A comprehensive approach was employed to retrieve relevant articles from PubMed.

###  Database Selection

 PubMed was chosen for its extensive coverage of biomedical and multidisciplinary literature.^32^ It was deemed suitable to capture a wide range of perspectives on the War on Gaza respective authors’ opinions, and humanitarian issues.

###  Search Strategy and Paper Identification

 A meticulous search strategy was crafted to capture the multidimensional aspects of the War on Gaza, encompassing political, scientific, and humanitarian dimensions. This approach combined relevant keywords and Medical Subject Headings (MeSH) terms, ensuring a comprehensive exploration of the topic. Boolean operators (eg, AND, OR) were utilized to refine the search process, allowing for the inclusion of diverse perspectives and facets. The selected terms—Gaza, Palestine, Genocide, War, Israel, Hamas, October 7, and Humanitarian—were designed to align with the study’s scope. Among these terms, the most effective search equation identified was:*“Gaza ”[Title] OR “Israel”[Title].*

 This equation was selected and applied in the search process. As an alternative to a systematic approach, the decision to use this refined search equation was made to effectively capture a substantial number of papers addressing the War on Gaza, while ensuring that no significant publications were overlooked. This strategy aimed to strike a balance between precision and comprehensiveness, allowing for a focused yet inclusive exploration of relevant literature.

 The most recent search conducted on October 7, 2024, to ensure the inclusion of the latest data. Non-English edited records were removed before screening. Duplicate papers were initially identified and removed using the Zotero library.

###  Screening and Exclusion Criteria

 Screening using titles and abstracts encompassed retrieved abstracts published in peer-reviewed academic medical journals between October 7, 2023, and October 7, 2024, whose contents pertain to the 2023-2024 War on Gaza ensuring that the review captures the most relevant literature. Papers were excluded at three levels: when the title/abstract was not relevant, the full text was not retrieved, or the contents did not directly address the study scope during the specified timeframe. Before concluding that the full text was not retrieved, several search attempts were made. These included contacting the corresponding author by email and checking their personal page on ResearchGate, if available.

###  Data Extraction and Charting Process

 Relevant data from each selected paper were systematically extracted using a pre-tested Excel data charting form. This form included fields for authorship, journal altmetrics, publication date, publication type, topics covered, and lexical fields. To identify terms for the lexical fields, a preliminary analysis was conducted using a sample of ten randomly selected papers, each independently reviewed by three authors *(EE, CBM, MB in the authors’ list).* A consensus approach was used to retain terms that addressed the political opinions and humanitarian aspects of the War on Gaza, such as genocide, violations of international humanitarian law, and impact on vulnerable populations. For certain terms, synonyms, such as “doctors,” “professionals,” or “healthcare workers,” were also sought to ensure thoroughness. Data extraction included the frequency and order of occurrence of these terms. The data charting form was refined based on this pre-test to ensure consistency and accuracy during the full review process.

 The most recent impact factors and ranking (in terms of quartile) of each journal were searched via the journal citation reports, respectively.^33^

###  Definitions

 Papers’ opinions and positions were classified as “pro-Gaza,” or “pro-Israel” when they met at least one of the following criteria: (*i*) Condemning the actions of one party while not condemning the other, and/or (*ii*) Justifying humanitarian crimes committed by one party, and/or (*iii*) Focusing predominantly on the burden of war, either humanitarian or medical, of one party but not the other.

 Papers’ opinions and positions were classified as “Neutral” if they did not meet the above criteria, and instead, provided a balanced view, addressing the actions and suffering of both parties without showing clear favoritism. This classification was used to systematically analyze and categorize the diverse scholarly perspectives on the War on Gaza.

 The terms “Pro-Gaza” and “Pro-Israel” were chosen for several reasons. These terms are concise, widely recognized, and commonly used in both academic and media discourse, ensuring accessibility for a broad readership. Their simplicity and repetition throughout the manuscript contribute to clarity and ease of reading. Furthermore, these terms allow for neutral, descriptive categorization of the literature, focusing on lexical and narrative distinctions rather than expressing subjective or ideological judgments, which is essential for maintaining objectivity in the analysis.

###  Quality Assurance

 To ensure the inclusion of high-quality and reputable sources, a rigorous quality assessment process was implemented. The review was conducted by a team with expertise in various fields of medicine and scientific writing, promoting a comprehensive evaluation of the publications. Several key components were incorporated into the process to minimize bias and maintain rigor:

####  Collaborative Review Process

 Each publication was independently evaluated by two authors (*EE and CBM in the authors’ list*) to ensure consistency and reduce subjectivity. In cases of discrepancies, a third reviewer (*MB in the authors’ list)* was involved to reach a consensus and make the final decision.

####  Diverse Academic Perspectives

 The interdisciplinary composition of the review team, with expertise across different academic fields, facilitated a broader understanding of the publications.

####  Standardized Methodology

 A structured protocol was followed, which included clearly defined criteria and a pre-established Excel data extraction form to ensure uniformity in data collection. This approach was designed to ensure that personal characteristics, such as nationality or religion, of authors did not influence the assessment, minimizing both intra-observer and inter-observer variability and keeping the focus on the content of the publications rather than irrelevant personal attributes.

###  Data Analysis

 To provide a comprehensive understanding of the studies included in the review, various bibliometric and linguistic patterns were analyzed. This involved extracting key characteristics from each paper, as well as analyzing the lexical fields and publication trends to better understand the political discourse surrounding the War on Gaza. Additionally, the publications were contextualized in relation to ongoing events, highlighting how the publications correlate with key moments in the conflict. The following steps were taken during the data analysis:

####  Papers Information

 Data for each article included in the review, such as author names, publication titles, country of affiliation, and journal names, were reported. Additionally, the frequency of key terms (eg, Gaza, occupation, doctors, hospitals) used in each article was recorded, with the counts of studied terms presented for each article. The retrieved papers were initially divided into three groups based on their respective stances: pro-Gaza, pro-Israel, or Neutral according to the defined categories.

####  Study Characteristics

 The characteristics of the included papers were categorized based on their stance (pro-Gaza, pro-Israel, or Neutral). A comparative analysis was conducted for journal names, altmetric rankings, publication types (eg, original research, correspondence, editorials), author affiliations, and the respective terms of lexical fields. For each analyzed category, counts, percentages, and statistical significance (*P* values) were derived from the comparisons.

####  Geographical Distribution of Authors

 The geographical distribution of first authors’ affiliations was examined to highlight disparities between pro-Gaza and pro-Israel stances. The number of studies affiliated with countries such as Palestine, Israel, the United Kingdom, and the United States was plotted to visualize the global perspectives represented in the sample.

####  Impact and Influence of Journals

 The distribution of studies across high-impact journals, such as *The Lancet* and *BMJ*, was assessed. This comparison also examined the proportion of pro-Gaza and pro-Israel papers published in these journals.

####  Publication Trends

 To examine the temporal evolution of the publications, weekly trends in publication volume were charted. These trends were complemented by data on Palestinian casualties, key events in the Gaza conflict, and the evolution of humanitarian conditions, such as water and food shortages and attacks on healthcare infrastructure. The data on these events, including the weekly cumulative number of Palestinians killed in Gaza, injured, and displaced Palestinians, were retrieved from updates by the UN Office for the Coordination of Humanitarian Affairs.^34^

####  Multivariate Analysis of Lexical Fields

 A multivariate analysis was performed to identify terms most strongly associated with pro-Gaza and pro-Israel positions. The terms independently associated with each position, along with odds ratios (ORs) and 95% confidence intervals, were charted to visualize linguistic differences between pro-Gaza and pro-Israel papers, highlighting the distinctive lexical choices.

###  Statistical Analyses

 Statistical analyses were conducted using SPSS software. For comparisons between groups, categorical data were reported as frequencies and percentages, while continuous data were represented by means and interquartile ranges. The student *t *test or the Mann-Whitney U test was used for continuous variables, and the chi-square test or Fisher’s exact test was applied for categorical variables, as appropriate. A *P* value <.05 was considered statistically significant. Binary logistic regression multivariate analysis was carried out using a manual stepwise backward elimination process. This included variables that demonstrated a significant association with the papers’ stances in the univariate model, with a *P *value <.2. ORs were calculated to estimate the strength of associations. This approach ensured a comprehensive assessment of the magnitude and relevance of observed differences.

 To ensure the validity of the statistical tests, we assessed the assumptions of normality and homogeneity of variance. Normality was tested using the Shapiro-Wilk test for continuous variables, and homogeneity of variance was assessed using Levene’s test. In cases where the assumptions were violated, non-parametric alternatives were applied: the Mann-Whitney U test or Wilcoxon test for continuous variables, and the Fisher’s exact test or the chi-square test for categorical variables, depending on sample size and expected frequencies. Data transformations were applied when appropriate to address non-normality.

 No missing data were encountered in the dataset, ensuring the reliability of statistical inferences drawn from the analysis.

## Results

###  Studies Flow Diagram

**Figure 1:**
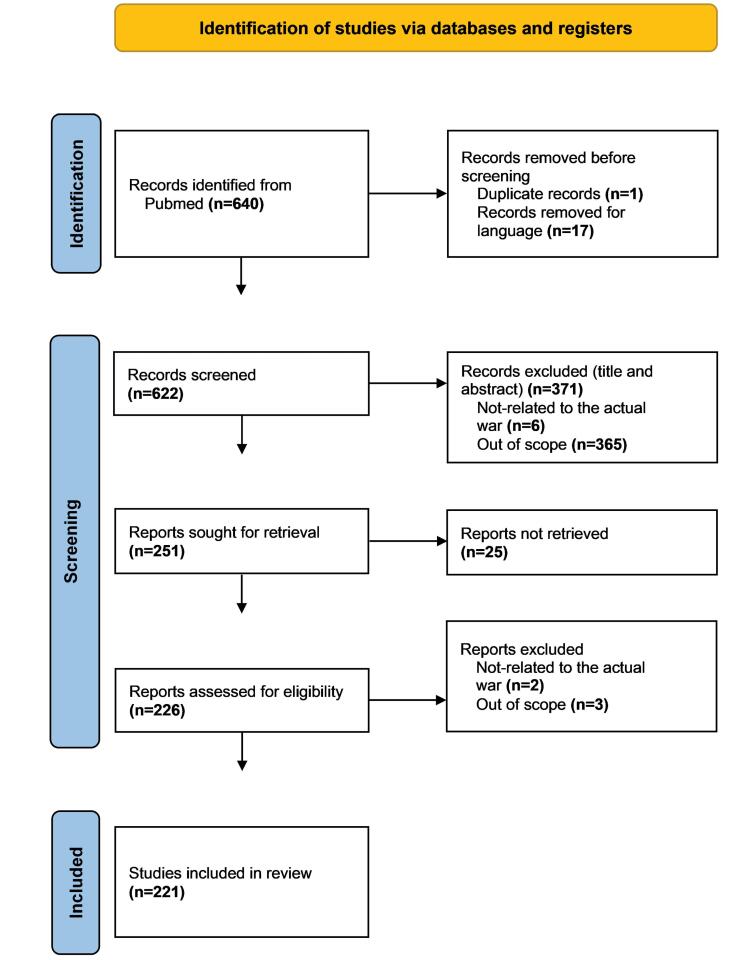
Flow Diagram Displaying the Identification, Screening, and Inclusion of the Papers Published From October 7, 2023, to October 7, 2024, Reporting Scholars’ Opinions About the 2023-2024 Gaza Genocide.

 The search strategy yielded 640 articles from PubMed. After removing duplicates and screening for relevance based on inclusion and exclusion criteria, 221 articles were included in the review (Supplementary file 1). Figure 1 illustrates the flow diagram detailing the selection process.

###  Characteristics and Bibliometric Analysis of the Included Papers According to Their Identified Positions Toward the War on Gaza 

Supplementary file 1 presents the individual characteristics and key charted data from the 221 papers included in the War on Gaza scoping review. There was a diversity of author opinions regarding the War on Gaza. While 25 (11.3%) studies exhibited Neutral position, 126 (57%) took a pro-Gaza stance, and 70 (31.7%) presented a pro-Israel perspective (Table 1).

**Table 1:** Compared Bibliometric Univariate Analysis of Studies Included From October 7, 2023, to October 7, 2024, Categorized According to Pro-Gaza (n = 126) or Pro-Israel (n = 70) Authors’ Positions

	**Pro-Gaza (n = 126)**	**Pro-Israel (n = 70)**	***P*** ** Value**
**Papers Characteristics**			
Journals			
Nature	4 (3.2)	2 (2.9)	.000
Lancet	39 (31.0)	17 (24.3)	
JAMA	4 (3.2)	6 (8.5)	
BMJ	29 (23.0)	3 (4.3)	
IMAJ	0	5 (7.1)	
Others	50 (49.7)	37 (52.9)	
Journals altmetrics			
Quartile 1	87 (73.1)	48 (71.6)	.028
Impact factor	54.6 ± 45.3	34.1 ± 41.9	.002
Country of the first author's affiliation			
Palestine	18 (14.3)	0	.000
Israel	0	46 (65.7)	
UK	32 (25.4)	5 (7.1)	
USA	19 (15.1)	11 (15.7)	
Others	57 (45.2)	8 (11.4)	
Number of authors/Paper	3.43 ± 5.1	3.7 ± 2.9	0.638
Publication type			
Original/Review	12 (9.5)	27 (38.6)	.000
Correspondence/Letter/Editorial	93 (73.8)	40 (57.1)	
News	21 (16.7)	3 (4.3)	
Papers’ topics			
Political opinions and beliefs	26 (20.6)	25 (35.7)	.000
Humanitarian issues	90 (71.4)	12 (11.8)	
Specific medical topics	7 (5.6)	15 (21.4)	
Psycho-social	3 (2.4)	18 (25.7)	
**Lexical Fields**			
**Addressed Themes**			
Papers’ title words			
Gaza	105 (83.3)	10 (14.3)	.000
Israel	8 (6.4)	49 (71.0)	.000
October 7			
Early reported in the text	15 (11.9)	48 (68.6)	.000
Condemnation	12 (9.5)	53 (75.7)	.000
Israeli military action			
Early reported in the text	38 (30.2)	2 (2.9)	.000
Condemnation	68 (54.4)	3 (4.3)	.000
Humanitarian issues			
IHL report	48 (38.1)	19 (27.1)	.157
IHL violation	50 (39.7)	18 (25.7)	.123
Individual displacement	57 (45.2)	12 (17.4)	.000
WHO	55 (43.7)	4 (5.7)	.000
UN	69 (54.8)	9 (12.9)	.000
Genocide			
Genocide/Punishment	30 (23.8)	9 (12.9)	.163
Cease-fire call	57 (45.2)	6 (9.5)	.000
Genocide/Punishment/Cease-fire call	72 (57.1)	13 (18.6)	.000
Socio-political background			
Occupation *(referring to the Israeli occupation of Palestine)*	36 (28.6)	3 (4.3)	.000
Antisemitism	6 (4.8)	7 (10.0)	.229
Word Count			
Gaza	13.17 ± 12.57	4.49 ± 4.81	.000
Israel	5.68 ± 8.32	12.73 ± 12.02	.000
Palestine	4.47 ± 8.01	1.84 ± 4.57	.004
Hamas	0.97 ± 1.70	3.69 ± 4.47	.000
Terror	0.14 ± 0.5	4.17 ± 6.60	.000
Human	4.56 ± 6.04	1.8 ± 2.59	.000
Hospitals	4.78 ± 7.89	3.86 ± 11.99	.517
Health	11.54 ± 13.86	6.87 ± 12.66	.021
Doctors/HCP	2.56 ± 4.7	3.71 ± 15.31	.440
Aid	1.56 ± 2.67	0.44 ± 1.24	.000
Medications	2.47 ± 4.04	0.45 ± 1.251	.000
Water-food-energy-sanitation	6.98 ± 13.96	1.11 ± 3.10	.000

Abbreviations: BMJ, British Medical Journal; HCP, healthcare professional; IHL, international humanitarian law; IMAJ, Israel Medical Association Journal; JAMA, Journal of the American Medical Association; UN, United Nations; WHO, World Health Organization. Values are presented as either No. (%) for counts and percentages or as mean ± standard deviation for continuous variables.

###  Compared Bibliometric Univariate Analysis

**Figure 2:**
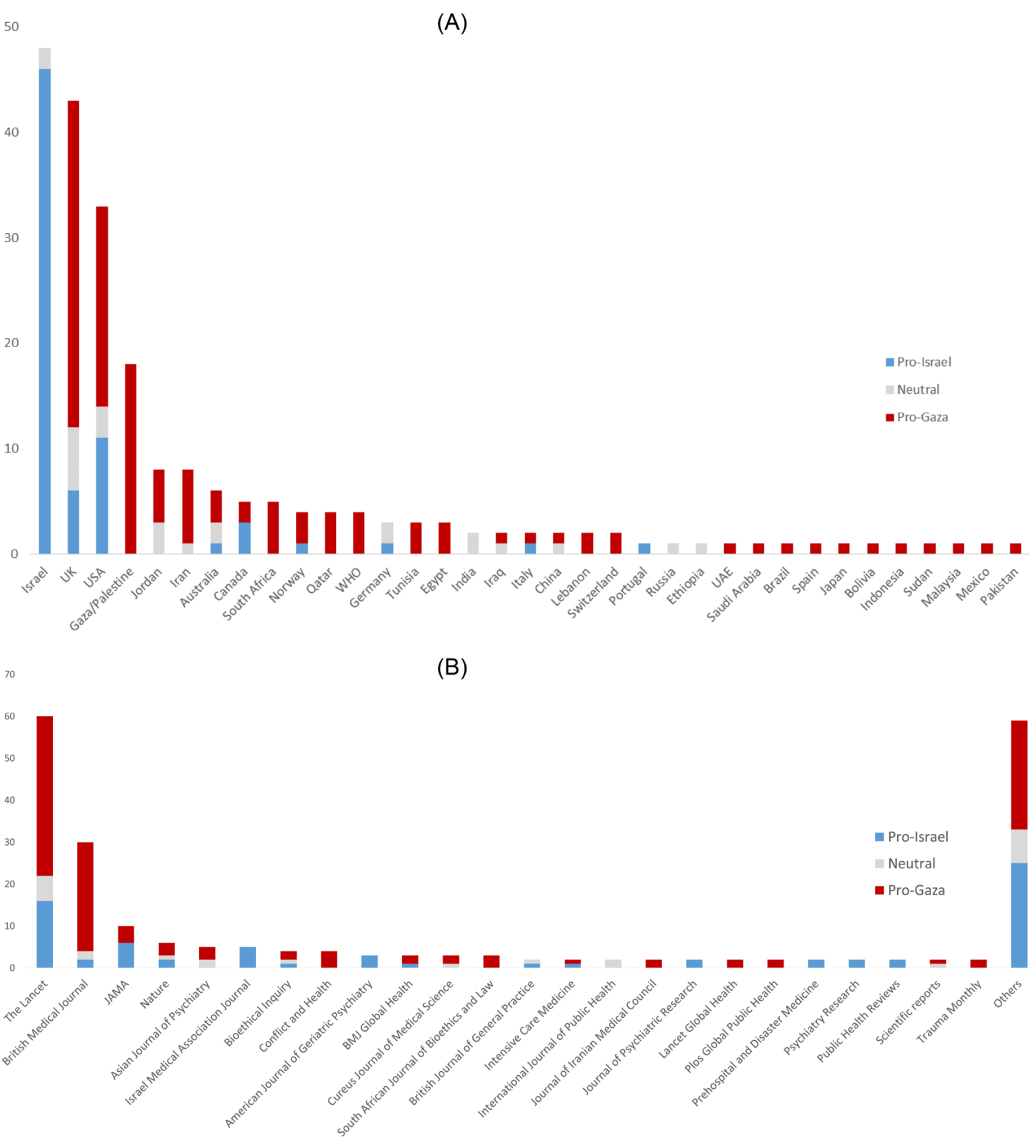
Publications’ Distribution (n = 221 Included in the Bibliometric Analysis) Within the War on Gaza From October 7, 2023, to October 7, 2024, According to the Affiliation Country (A), the Journal (B), and the Papers’ Analyzed Opinions (Pro-Gaza, Pro-Israel, Neutral).

 Papers were published by authors affiliated with 34 countries (Figure 2A). Pro-Israel papers originated from nine countries, primarily Israel, the United Kingdom, and the United States. Pro-Gaza papers were affiliated with 29 countries, with the most common affiliations being the United Kingdom, Palestine, the United States, Iran, and South Africa. The 221 analyzed papers were published in 83 scientific peer-reviewed journals. A high proportion of the publications 176 (79.7 %) were in the form of correspondence, editorials, opinions, or news articles. Two main highly ranked journals encompassed half the publications: *The Lancet* (n = 60, pro-Gaza/pro-Israel/Neutral, 39/17/6) and *BMJ* (n = 30, pro-Gaza/pro-Israel/Neutral, 29/3/2) (Figure 2B). Notably, Richard Horton, editor-in-chief of *The Lancet,* has authored three “Pro-Israel” articles, a noteworthy observation considering his influential role in shaping medical discourse (Supplementary file 1).

Table 1 provides a comparative analysis of the bibliometric data extracted from the studies, categorized according to the authors’ positions, whether pro-Gaza or pro-Israel. The main findings can be summarized as follows:

- Pro-Gaza stances represented two-thirds of the reviewed papers.
- Pro-Gaza papers were published in more impacted journals and exhibited better altmetrics.
- While pro-Gaza authors were affiliated with various international institutions, pro-Israel authors were mainly affiliated with Israeli Institutions.
- Both groups predominantly published short papers (Correspondence, Letters, Editorials), but pro-Israel authors produced significantly more original papers.
- Pro-Israel authors more frequently published political opinions and papers on specific medical topics mainly psychological impact. Conversely, pro-Gaza papers focused more on humanitarian issues, called for a cease-fire, and characterized the crime of genocide.
- The lexical fields used were distinctly different, with pro-Gaza papers exhibiting humanitarian depth and pro-Israel papers emphasizing self-defense.

###  Trends of opinions discussed in the different papers

**Figure 3:**
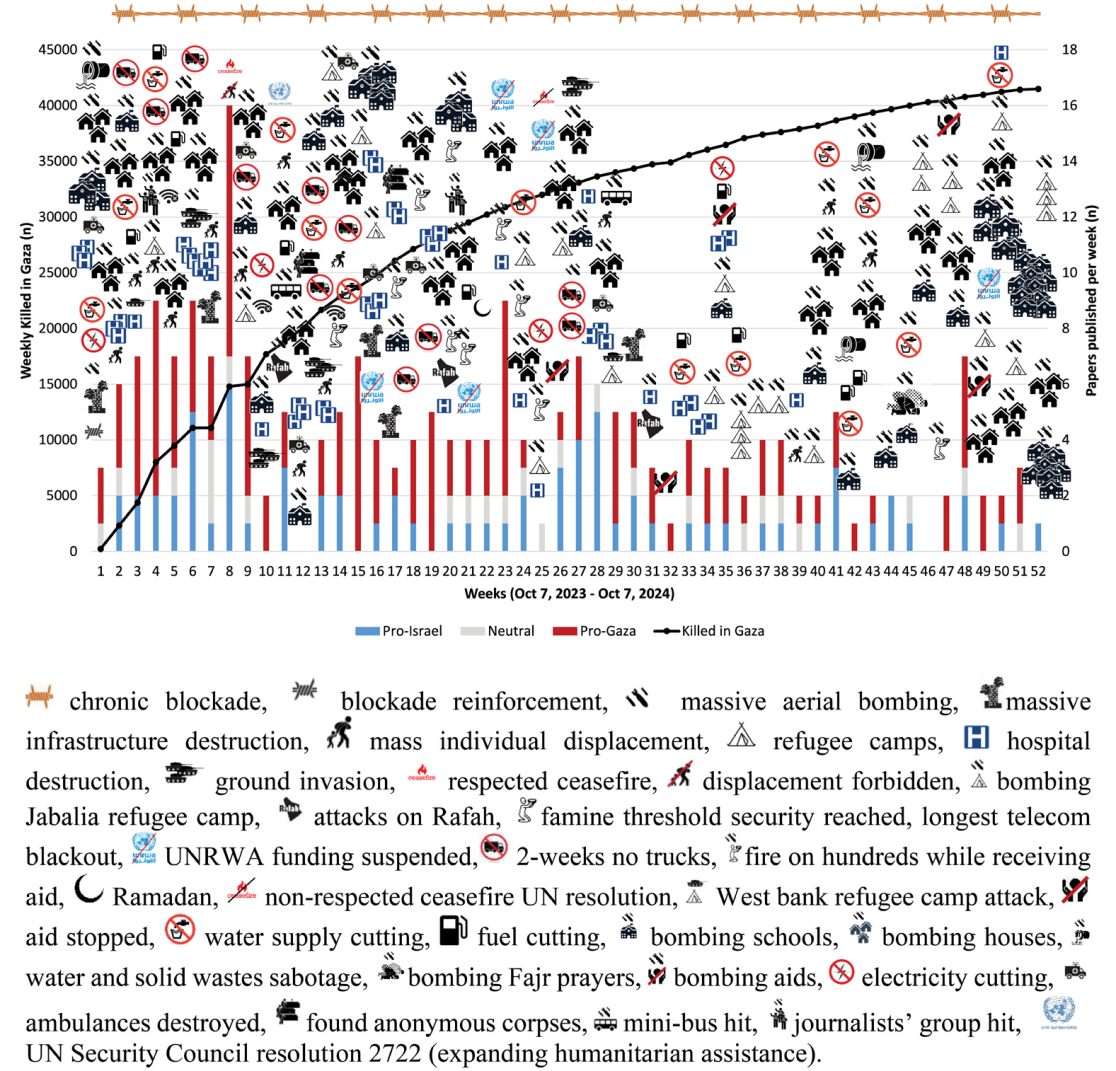
Weekly Publications’ Trends (n = 221 Papers Included in the Bibliometric Review) Within the War on Gaza From October 7, 2023, to October 7, 2024, and Distribution According to the Papers’ Analyzed Opinions (Pro-Gaza, Pro-Israel, Neutral). Weekly cumulative number of Palestinians killed in Gaza (black curve) according to the UN Office for the Coordination of Humanitarian Affairs^34^ and main War on Gaza events are plotted to search for explanatory factors. Abbreviations: UN, United Nations; UNRWA, UN Relief and Works Agency.

 During the 52 weeks of the study, the flow of publications on the War on Gaza was particularly intense during the first two months, before stabilizing at an average of five articles per week, with a predominance of pro-Gaza opinions. To understand this dynamic, we explored the evolution of the situation on the ground and the increase in the weekly cumulative number of Palestinian citizens killed (Figure 3).

 The intensified blockade from October 9 exacerbated water, electricity, and food shortages. Aerial bombings, ground invasions, malfunctions and destruction of healthcare infrastructure, restrictions and prohibitions on humanitarian aid, ceasefires that were not respected, bombings of refugee camps and schools, and bombings of civilians seeking food aid occurred daily and weekly. Consequently, over these 52 weeks, 41 965 Palestinian citizens were killed, 97 590 were injured, nearly 1.9 million people were massively displaced, and a famine threshold was reached by the 14th week (Figure 3).^35^

 Attacks on the healthcare system, totaling 516 attacks, killed 765 healthcare professionals (HCPs) and injured 990. Additionally, 110 healthcare structures were affected, including 32 of Gaza’s 36 hospitals, with 75/137 (56.8%) rendered non-functional and 115 ambulances damaged (Figure 3).^35^

###  Multivariate Analysis of the Lexical Fields Used in the Reviewed Papers

 The multivariate analysis identified terms independently associated with the pro-Gaza position, revealing a characteristic lexical field (Table 2). This field included the terms “Gaza,” prominently displayed in the title, “occupation” to emphasize the historical context of the Palestine-Israel conflict, “genocide” or “collective punishment” to describe the war crimes perpetrated by Israel against the Palestinian people, and “ceasefire appeals.” Conversely, the pro-Israel lexical field featured terms like “Israel” in the title, references to “October 7” prominently displayed and condemned as early as in the abstract or title, and “Hamas,” often described as a terrorist organization (Table 2).

**Table 2:** Multivariate Analysis of Terms Independently Associated Respectively With Pro-Gaza (n = 126) and Pro-Israel (n = 70) Positions

	**ORs [95% Confidence Interval]**	***P***
Pro-Gaza position		
Gaza isolated in the title	25.93 [10.94 to 61.49]	.000
Occupation *(referring to the Israeli occupation of Palestine)*	5.22 [1.42 to 19.17]	.013
Genocide/punishment/ceasefire	2.69 [1.11 to 6.53]	.029
Pro-Israel position		
Israel isolated in the title	41.74 [4.87 to 357.55]	.001
October 7 early reported in the text	4.61 [1.95 to 10.90]	.000
Hamas	5.81 [2.16 to 15.68]	.001

Abbreviation: ORs, odds ratios.

## Discussion

 The present scoping review identified 221 papers published within the first year of the War on Gaza. Two-thirds of the papers exhibited pro-Gaza stances, were published in more impactful journals, had authors with worldwide affiliations, addressed significantly more humanitarian issues, and more frequently called for a ceasefire, and/or decried the genocide.

 Beyond the involvement of scientists in this War on Gaza, this review distinctively identified and highlighted specific lexical fields associated with each opinion holder. These lexical choices likely stem from their origins, affiliations, the environments in which they have evolved, and from a historical reality that is often manipulated and instrumentalized. The present scoping review and bibliometric analysis is another demonstration of the so-called “war of words,” probably a consequence of the arrangements and distortions of this reality.^21,23^

 While emphasizing the rigor of our study selection and data processing, we acknowledge that our pro-Palestinian positionality may have shaped data interpretation but not data analysis. We see positionality not as a limitation but as an inherent part of research, requiring awareness and reflexivity. By recognizing our perspective transparently, we present it as a critical lens rather than a bias.

###  Trends and Characteristics of Scholarly Reactions to the 2023-2024 War on Gaza

 The present scoping review and bibliometric analysis highlights the active involvement of scholars in the political and humanitarian issues concerning the 2023-2024 War on Gaza. Despite the escalating atrocities against Palestinians, there has not been a notable increase in the number of published articles or the depth of their content. Our study identified a stable trend, with pro-Gaza publications making up two-thirds of the weekly output compared to pro-Israel publications. This predominance indicates the scholarly community’s tendency to support the Palestinian cause. However, the actual ratio may be higher, as many scholars might face professional, social, and editorial pressures that prevent them from openly expressing their views.^36^ Indeed, censorship against pro-Palestinian publications, even in the field of health, is not a recent phenomenon.^37^ Long before the current War on Gaza, the Palestinian narrative was often either censored or accepted only when an Israeli counter-version was simultaneously co-published, while the Israeli perspective often remains unchallenged, creating an illusory “balance” that overlooks profound power imbalances.^38^ The retraction of a publication in The Lancet warning about the challenges of Coronavirus Disease 2019 on Gaza’s healthcare system, weakened by years of Israeli violence,^37,38^ and another in American Scientist supporting the Boycott, Divestment, and Sanctions campaign,^39,40^ are perfect examples. Other publications have faced aggressive attacks or defamation.^41-43^ During the current war, scientists have faced significant pressures preventing them from publishing freely.^36,44^ A notable instance is the collective action of 148 doctors who petitioned the General Medical Council to issue guidelines on “acceptable” social media postings for doctors regarding the War on Gaza.^45^ This call for regulation underscores the sensitivity and high stakes associated with public commentary on the War on Gaza.^45^ In Australia, the situation was similar, where several doctors were reported to the Australian Health Practitioner Regulation Agency following their public comments on the War on Gaza. A medical organization even issued a warning, advising doctors to “carefully consider” any public comments on the war.^44^ An editor-in-chief of an open-access science journal has been dismissed following a controversy over his social media posts about the War on Gaza.^44^

 Beyond the scientific narrative, which is ultimately only a consequence of a policy described by historians as a “systematic epistemicide of Palestinian history,”^25,46^ publishing to assert the Palestinian perspective in any discipline has become a struggle against systematic censorship and a true act of resistance.^36,47^ Israeli efforts to discredit and sabotage Palestinian knowledge production and history are classic strategies employed by settler colonialism to uphold their narrative.^48^ In response, colonized societies find themselves obligated to rebuild their narratives anew, often adopting those of their colonizers, as their own histories have been systematically dismantled.^46^ As a consequence, scholars advocate for a nuanced and indigenous-centered approach, thereby contributing to a more accurate and empowering historiography highlighting both the ongoing colonial violence and the oppressed resilience.^25^ This epistemic erasure is not merely a contemporary phenomenon but is deeply rooted in the historical trajectory of Zionist colonization, and it can be traced back to the late 19th century, with the emergence of the first Zionist movements, whose objectives were to create a country for “a nation” and the establishment of the first Zionist settlements on Palestinian land, whose existence is undeniable but whose history has been deliberately ignored.^49,50^ The Balfour Declaration of 1917 exacerbated the injustice against Palestinian citizens by neglecting their political rights and favoring Zionist ambitions.^51^ Increasing clashes erupted between Jewish migrants, who came in large numbers from various countries, encouraged by Western powers, and Palestinian citizens, defending their lands, lifestyle, homes, and rights, which were being usurped by Zionist settlers.^50,51^ The 1948 year marked a major turning point in the Zionist project and in the lives of Palestinians.^27^ That year signaled the beginning of a struggle for Palestinians to exist as a people and even to be recognized as such.^52^ The “Nakba,” which literally means “catastrophe or disaster,” not only refers to the proclamation of the Zionist state on Palestinian lands but also to the cascade of injustices that followed this crime against a people expelled from their ancestral land.^53,54^ This included the clear collapse and disintegration of their social structure, the loss of their identity, the displacement of approximately one million refugees to Arab countries, the West Bank, and the Gaza Strip, the destruction of 418 Palestinian villages, and the brutal massacres, assassinations, and acts of terror perpetrated by Zionist groups.^52^ The Nakba is not only a calamity for Palestinians but also a stain of shame on the world’s conscience.^52^ It stands as evidence of the absence of universal justice and the failure of what is called the international community to resolve the issue for over seventy years.^52^ Since this pivotal moment, the West Bank was under Jordanian control, and the Gaza Strip under Egyptian military administration, both completely isolated from the rest of the Palestinian territories.^51^ Their occupation by Israel following a military action in June 1967, conducted in violation of the *jus ad bellum* rule,^55^ prohibiting the use of force, and the adoption of UN Resolution 242,^56^ which essentially expresses the idea of two states, made the West Bank and Gaza the sole geographical framework for Israeli-Palestinian negotiations. When Israel pretended to negotiate the Palestinians’ right to become a state, it was only to concede a caricature: a demilitarized enclaved power, scattered over a fragmented territory, with reduced access to its natural resources.^57^ However, the right of Palestinians to self-determination has the status of a *jus cogens* norm.^58^ It is not a constitutive right that can only arise from its recognition by Israel.^59^

 In this bibliometric analysis, it is not surprising to find that pro-Israel papers were authored primarily by authors affiliated with institutions in Israel or the United States. In contrast, pro-Gaza authors were affiliated with institutions worldwide, reflecting the global impact of the 2023-2024 War on Gaza on international public opinion and an unprecedented engagement with the Palestinian cause. This widespread support is underscored by numerous global protest movements, notably the Students for Palestine movement, which fervently advocates for justice for Palestinians.^60^ Several publications were categorized as neutral because their authors refrained from taking a stance on either side. Neutrality often presented as an attempt to remain unbiased and as an imperative of the scientific mind, generally favors simply the dominant discourse. This approach is particularly problematic when historical truths are biased in favor of colonizers and disadvantage the colonized, especially during periods of severe oppression and the extermination of a people, as it leaves the root causes of the conflict unaddressed and contributes to a narrative that normalizes the status quo.^61^ Such positions, while seemingly rooted in a commitment to human values, ultimately serve to sustain the very injustices they ostensibly seek to mitigate, thereby reinforcing the cycle of violence.^28^

 Our scoping review and bibliometric analysis revealed that almost all the publications 176 (79.7%) were in the form of correspondence, editorials, opinions, or news articles, which are more suited for sharing viewpoints. A small proportion of original articles, primarily pro-Israel, reported on medical issues resulting from the War on Gaza. Conversely, Palestinian researchers faced significant barriers in pursuing their scientific work or continuing clinical activities due to attacks on universities and hospitals and the arrest of doctors.^62-64^ Some authors described the targeting of medical professionals and facilities as part of a systematic genocide.^59^ This stark contrast clearly illustrates that while life continues relatively normally on one side, lives are being lost daily on the other.^62^ While most articles in our sample express support for Gaza, the fact that pro-Israel positions dominate original research articles highlights a significant power imbalance in academic publishing. Original articles are often considered more credible and impactful in the scientific community compared to correspondences or opinion pieces, which hold less weight. This disproportionate representation of pro-Israel narratives in such high-value academic outlets reflects broader power dynamics within both the academic and geopolitical spheres. The privileging of pro-Israel viewpoints in these scientific articles is not only indicative of how knowledge production is shaped by political forces but also underscores the role of academic institutions, often influenced by political and ideological ties, in determining what is considered “legitimate knowledge.”^65^

 Regarding the topics debated in the reviewed papers, pro-Gaza publications predominantly focused on humanitarian issues, while pro-Israel papers covered a balance of political and scientific topics, particularly the psychosocial collateral consequences of the war. The extent of the atrocities—including loss of life, infrastructure destruction, and starvation—explains the emphasis of pro-Gaza papers on the impact on the civilian population, even suggesting the extermination of future Palestinian lifestyles.^59^

 Regarding lexical fields, it is immediately noticeable that pro-Gaza and pro-Israel papers used the isolated terms “Gaza” or “Israel” in their titles, clearly indicating their stances. In this study, a notable pattern emerged in how the events of October 7 were reported and condemned. The frequent citation of October 7, condemned by both pro-Israel and pro-Gaza, has been more commonly used at the beginning of pro-Israel articles. In some cases, it has been used, such as by the Israeli government, to justify military actions against the Palestinians, and at times as a reference to the beginning of the atrocities committed in Gaza. Consequently, its frequent use in articles and its placement at the beginning, sometimes even required by editors,^66^ can influence readers’ opinions regarding the legitimacy of crimes against humanity committed against Palestinian civilians, thus giving an erroneous perspective of the origin of this conflict, which dates back not to October 7, but much earlier, to the Israeli occupation of Palestine.^52^ The term “Hamas” appears significantly more frequently in pro-Israel articles compared to pro-Gaza ones, aligning with the narrative of self-defense against a “designated terrorist organization.” Conversely, the term “occupation” is predominantly used in pro-Gaza articles, emphasizing the narrative of prolonged oppression and the illegitimacy of the Israeli presence in Palestinian territories. These distinct terms serve as defining lexical fields to identify the stance of respective papers, as demonstrated by multivariate analysis. The strikingly high ORs of the terms “Gaza” (OR: 25.93) and “Israel” (OR: 41.74) when displayed early in the title underscore their pivotal role in signaling the alignment of the narrative with pro-Gaza or pro-Israel stances, respectively. This prominent positioning reflects the authors’ intent to establish the core focus and orientation of their discourse from the outset. The remaining terms identified in the analysis, each with consistently high ORs, further define the unique characteristics of these narrative frameworks. Pro-Gaza narratives are shaped by terms like “occupation” (OR: 5.22) and statements such as “genocide,” “collective punishment,” and “ceasefire appeal” (OR: 2.69), highlighting themes of long standing systemic injustice and calls for humanitarian intervention. In contrast, pro-Israel narratives emphasize immediate security concerns, with early references to “October 7” (OR: 4.61) and frequent mentions of “Hamas” (OR: 5.81), framing the narrative through a lens of immediate security threats and the identification of perceived adversaries. The self-defense narrative, often invoked to justify actions in the region, unfortunately obscures the ongoing violation of fundamental human rights initiated by “the Israeli military occupation of Palestine,” whose colonial expansion has never ceased despite its illegality, was formalized by Israel in its 2018 Basic Law, enshrining the development of Jewish settlements as a fundamental value of Israeli society.^67,68^ The settlers are even encouraged and armed by the Israeli state itself. Due to its duration and the practices deployed, the occupation is a pretext for an annexation project.^69^ This project formalized *de jure* for Jerusalem, self-proclaimed “the unified capital of Israel,”^70^ is being implemented *de facto* for the West Bank.^71^ As for Gaza, since Israel’s 2005 withdrawal from the Gaza Strip, and has imposed a blockade since 2007,^72^ amplifying the precarious living conditions and the injustice inflicted on Palestinian people.^72^ Israel has launched twelve retaliatory military offensives against this Palestinian territory in 17 years, resulting in approximately 4000 deaths.^72^ While Palestinian uprisings are deeply rooted in the quest for self-determination, independence, and responses to violations of basic rights, including freedom of movement, unjustified arrests, apartheid policies, and the right of return,^73^ “self-defense narrative” often invoked to justify Israeli actions, reduces any form of Palestinian resistance to “terrorism.”^51^ Yet Palestinian resistance is recognized by the UN (General Assembly, 1970) as “legitimate by all means at their disposal.”^74,75^

 Throughout the one-year perpetrated atrocities, 23.2% of the pro-Gaza scholars mentioned in the present scoping review denounced the genocide, with some even crying out about it from the very beginning.^76^ In March 2024, a report issued under the auspices of the UN by expert Francesca Albanese concluded that there are reasonable grounds to believe that the threshold indicating Israel’s commission of genocide is met.^77^ Within the analyzed papers, three key pillars emerged as defining characteristics of the genocide. The blockade, a key pillar of this genocide,^10^ has intensified since October 9,^78^ leading to widespread starvation. In our study, terms related to starvation or its equivalents *(food, water)* appeared significantly more frequently in pro-Gaza papers compared to pro-Israel papers (6.98 ± 13.96 vs 1.11 ± 3.10, *P* =.0001). The World Food Program declared that the famine threshold was reached by week 14.^79^ Israel has weaponized starvation by blocking aid and closing crossings.^80^ The systematic destruction of Gaza’s health system, including the targeting of hospitals and health professionals,^15^ represents another pillar of this genocide.^81^ This was frequently highlighted in the reviewed papers through various terms such as *hospitals, health, doctors/HCP, World Health Organization (WHO), and medication*. The Israeli Army’s destruction of cities has displaced nearly two million people into refugee camps, ravaging all aspects of life in Gaza and forming the third pillar of the genocide. Figure 3 illustrates the cumulative weekly destruction, systematically targeting all aspects of life. Time-series satellite remote sensing conducted between October 2023 and March 2024 reveals a gradual increase in war damage across the Gaza Strip, with 58.4% of residential and educational structures affected, further worsening the housing crisis and potential displacement of the population. Additionally, a 34.1% reduction in cultivated agricultural land poses a significant risk to food security.^82^ Over 42 000 citizens, mostly children, have been killed, with the UN Secretary-General calling Gaza a “graveyard for children.”^83^ Indirect deaths from starvation, lack of medical care, and infections remain uncountable.^76^ According to a Lancet paper based on similar recent conflicts, up to 190 000 or even more deaths could be attributed to the ongoing genocide in Gaza. It is estimated that half a million people will be lost by the end of the year.^76^ As Israel’s genocide in Gaza continues, Israeli settler colonialism expansion in the West Bank accelerates, with 3500 new illegal housing units approved in March. Settlers, often backed by soldiers, have ramped up attacks. Over 4555 Palestinians have been displaced, with 744 killed, including 164 children, and more than 6250 injured.^34^

 Throughout the year-long lasting War on Gaza, the international community has failed to stop Genocide. The UN Security Council issued weak resolutions for humanitarian pauses and aid delivery but lacked radical solutions to end the genocide.^84-86^ Ceasefire attempts and full Palestinian UN membership were blocked by American vetoes.^87^ The evident delays, inadequacies, and disregard for UN resolutions are clear signs of the international community’s complacency in the face of war crimes. This can be attributed to the persistent power asymmetry between former colonial powers and the colonized regions, along with the disproportionate influence of Western governments, particularly the United States. This contrasts strikingly with the swift and punitive actions taken against Russia in the Ukraine war.^88,89^

###  Academic Writing, Global Health, and Social Accountability Amidst the Palestinian Genocide in Gaza

 Globally, humanitarian crises now affect more people than at any other point in history.^90^ These crises have immense health impacts, yet the responses from scholars and humanitarian organizations are often weak and inadequate.^91^ In five weeks alone, the number of civilians killed in the Occupied Palestinian Territory was equivalent to almost 60% of the total global number of civilians killed in 2022, which was itself already the deadliest year since the Rwandan genocide in 1994.^92^

 Typically marginalized within the realm of global health, these crises should nevertheless be integrated into comprehensive approaches and strategies.^93^ This is especially crucial if we hope to achieve ambitious global health targets such as the WHO Sustainable Development Goals.^94^ Face to the countless attacks on health during this genocide, global health institutions failed to address the persistent violence and the blatant targeting of health facilities and healthcare workers.^95^ How and why have our global health institutions allowed the situation to deteriorate so drastically?^96^

 The evident inability of these institutions to counteract the intense violence and stranglehold of influential governments underscores the urgent need for global health to confront the realities of settler colonialism and its entanglement within the systems that uphold these colonial practices.^93^ This exemplifies the shortcomings of the “decolonizing global health” approach endorsed by global health institutions.^93^ It reveals the limitations of a strategy that is strong in rhetoric but weak in implementing a radical change to the entire system that supports and perpetuates settler colonialism.^97^ Decolonizing global health demands, a more radical strategy that goes beyond simple institutional reforms, aiming instead for a transformative overhaul of current structures.^98^

 Tackling this issue also requires a thorough reassessment of the existing knowledge politics in global health.^99^ The field is characterized by a conventional top-down “knowledge translation” model, primarily controlled by Global North institutions that act as gatekeepers of knowledge.^100^ This dominance results from the marginalization and suppression of local perspectives, particularly through academic censorship,^37^ especially from areas like Palestine.^101^ This approach involves dismantling the entrenched settler-colonial infrastructures and policies that perpetuate inequalities and reframing health interventions to center reparations, justice, and the lived realities of marginalized communities.^65,93^

 The considerable power and authority of medical institutions, if mobilized effectively, could have served as a pivotal force in reshaping the narrative around attacks on healthcare; however, the prevailing discourse often reflects the opposite, with some authors even asserting that such actions can be justified as self-defense.^101^

 As doctors rooted in human values, our responsibilities extend beyond clinical care to advocating for justice, human rights, and the well-being of vulnerable populations.^102-104^ In the context of the ongoing genocide in Gaza, it is imperative to use our scholarly skills to highlight the humanitarian crises faced by the Gazan population within the historical framework of settler colonialism.^93^ Our objective must go beyond merely documenting these atrocities; we must also urgently advocate for actions to alleviate suffering and uphold the dignity and rights of the Palestinian people, fulfilling our ethical duty to promote health, equity, and justice on a global scale.^91^ Through our writing and publishing, we engage in a significant “war of words,” shaping a historiography that deeply impacts an entire people.^26^ The mere selection and framing of words can significantly influence the understanding of the conflict and the legitimacy of the parties involved.

 For Palestinians, reconstructing their own history since the occupation, subjected to a true epistemicide by Zionist colonialism, is not merely a form of resistance but also a way to demonstrate to a “blind” world their right to exist as a people.^25,46^ By breaking the silence and embracing our social accountability, we can amplify marginalized voices and advocate for active global intervention, challenging the indifference that often accompanies claims of neutrality by scholars and international bodies.^61^

###  Strengths and Limitations

 This study on scholars’ reactions to the War on Gaza presents several key strengths. Firstly, it provides a comprehensive and nuanced understanding of the diverse perspectives held by scholars, addressing one of the most intensely mediated yet often silenced genocides. By incorporating a wide range of scholarly voices, the study ensures a balanced representation of opinions, thus enriching the discourse on the subject. Secondly, the methodology employed is “robust,” combining scoping review and bibliometric analysis with a thorough review of existing literature, which allows for a deep exploration of the underlying explanatory factors influencing scholars’ reactions. Additionally, the study’s relevance is underscored by its timeliness, addressing contemporary issues that are crucial for policy-makers, educators, and the global academic community. The findings not only contribute to the existing body of knowledge, but also provide actionable insights for future research and policy formulation, particularly in the realms of conflict resolution and academic freedom.

 Several limitations could be addressed. *First,* the search was confined to PubMed, which may not fully capture the breadth of scholarly discourse on this topic. While this approach ensured a manageable, human-driven analysis of bibliometric data, it may have introduced selection bias, as PubMed-indexed journals often prioritize certain thematic or regional perspectives. Additionally, this reliance on a single database may limit the generalizability of the findings, as it does not fully encompass perspectives from other regions or less-represented disciplines. Expanding the scope to include additional databases, such as Scopus, Web of Science, or regional and non-indexed journals, could enhance the comprehensiveness and diversity of future reviews. Employing advanced tools, such as artificial intelligence, may also facilitate the handling of larger datasets required for such an expanded approach. *Second,* the choice of keywords may have overlooked synonymous or contextually relevant terms, potentially restricting the breadth of articles analyzed and excluding studies that could have broadened the review’s scope. *Third,* while subjectivity is inherently unavoidable, extensive efforts were made to minimize its impact. This was achieved using *a priori* standardized definitions and pre-tested data charting forms, and by reaching consensual decisions whenever discrepancies arose.

## Conclusion

 This scoping review and bibliometric analysis has examined the diverse opinions and positions of scholars expressed through academic papers on the Palestinian Genocide in Gaza, highlighting the complex War of words surrounding this conflict. Pro-Gaza stance, significantly surpassing pro-Israel stance, was prominently represented worldwide. This position was reported by higher-impact journals, addressed more humanitarian issues, and frequently called for a ceasefire. By analyzing the lexical fields used, we have traced the historical and ongoing humanitarian issues intertwined with the chronic occupation of Palestinian territories. The analysis reveals that scholars’ positions on the War on Gaza cannot be dissociated from the broader context of its chronic occupation. This enduring occupation has been discussed in the analyzed papers, emphasizing the persistent struggle for justice. The large-scale and intense Israeli attacks on Gaza were rapidly documented to be what many described as the most characterized and widely mediated genocide, occurring with total impunity. Despite the calls for a ceasefire that persist beyond the study’s timeframe, these appeals have remained largely unheard. Meanwhile, starvation is increasingly affecting the survivors, dominating the immediate humanitarian landscape, while the chronic occupation continues to linger in the background. International bodies and global health organizations have unfortunately failed to adequately address this humanitarian crisis. This underscores the urgent need to revisit and strengthen the current decolonizing approach to global health.

## Ethical issues

 This study did not involve human or animal participants and therefore ethics approval is waived by the Institutional Ethics and Research Committee of the University Hospital Farhat Hached, Sousse, Tunisia. IORG 0007439 ERC 05 04 2024.

## Conflicts of interest

 Authors declare that they have no conflicts of interest.

## Transparency

 All authors affirm that this manuscript is an honest, accurate, and transparent account of the study being reported. No important aspects of the study have been omitted, and any discrepancies from the study as originally planned have been explained.

## Declaration of generative AI’s role in scientific writing

 The authors wish to disclose that an artificial intelligence tool (ie, ChatGPT 3.5) was utilized to enhance the clarity and coherence of the manuscript’s writing. The tool was utilized for language refinement purposes only, ensuring the text was clear and coherent without altering the scientific content or generating any new text.

## Patient and public involvement

 The present study focuses on academic publications and does not involve any direct interaction with patients or the public as participants.

## Data availability statement

 The data that support the findings of this study are available from the corresponding author, Mohamed Boussarsar, upon reasonable request.

## Supplementary files


Supplementary file 1. Characteristics of the Main Charted Data of the 221 Papers Included in the Scoping Review.
