# Supplementary material for: Scholarly Publications and Opinions Through 366-Day War on Gaza (2023-2024): A Scoping Review and Bibliometric Analysis
Source: Int J Health Policy Manag. 2025 Apr 14;14:8809. doi: 10.34172/ijhpm.8809 (PMC12089835; doi:10.34172/ijhpm.8809)
Supplement: Supplementary file 1 — Characteristics of the Main Charted Data of the 221 Papers Included in the Scoping Review. [file ijhpm-14-8809-s001.pdf]

Article title: Scholarly Publications and Opinions Through 366-Day War on Gaza (2023-2024): A Scoping Review and Bibliometric Analysis

Journal name: International Journal of Health Policy and Management

Authors' information: Emna Ennouri[1,2,3]¶, Mohamed Boussarsar[1,2,3]*¶, Chourouk Ben Mahfoudh[4], Khamis Elessi[5], Helmi Ben Saad[1,3,6]

[1]Faculty of Medicine of Sousse, University of Sousse, Sousse, Tunisia

[2]Medical Intensive Care Unit, Farhat Hached University Hospital, Sousse, Tunisia

[3]Research Laboratory "Heart Failure", Farhat Hached University Hospital, Sousse, Tunisia

[4]Faculty of Medicine of Tunis, University of Tunis El Manar, Tunis, Tunisia

[5]Evidence-Based Medicine Unit, Faculty of Medicine - Islamic University of Gaza, Gaza, Palestine

[6]Laboratory of Physiology and Functional Explorations, Farhat Hached University Hospital, Sousse, Tunisia

***Correspondence to:** Mohamed Boussarsar, hamadi.boussarsar@gmail.com

¶ Both authors contributed equally to this paper.

Citation: Ennouri E, Boussarsar M, Ben Mahfoudh C, Elessi K, Ben H. Scholarly publications and opinions through 366-day war on Gaza (2023-2024): a scoping review and bibliometric analysis. Int J Health Policy Manag.2025;14:8809. doi:10.34172/ijhpm.8809

Supplementary file 1. Characteristics of the Main Charted Data of the 221 Papers Included in the Scoping Review.

Author	Author number	Topic	Title	Journal	Publication Date	Publication Type	Country of first author affiliation	Journal IF	Journal Q	Position	Gaza In Title	Israel In Title	Occupation *(referring to the Israeli occupation of Palestine)*	October 7 Citation	Genocide
**Elisabeth Mahase**[1]	1	Humanitarian	Israel and Gaza: MSF calls for end to "indiscriminate violence and collective punishment	BMJ	13-Oct-23	News	UK	93.6	1	pro-Gaza	0	0	1	1	0
**Hina J Shahid**[2]	2	Humanitarian	The healthcare community must approach the violence in Israel and Gaza with inclusive compassion	BMJ	13-Oct-23	Correspondence / Letter / Editorial	UK	93.6	1	Neutral	0	0	4	1	1
**Khamis Elessi**[3]	1	Humanitarian	Save Gaza residents from imminent catastrophe	The Lancet	13-Oct-23	Correspondence / Letter / Editorial	Gaza / Palestine	98.4	1	pro-Gaza	1	0	2	0	1
**Elisabeth Mahase**[4]	1	Humanitarian	Gaza: Israel's hospital evacuation orders are "death sentence for the sick" say WHO	BMJ	16-Oct-23	News	UK	93.6	1	pro-Gaza	0	0	0	0	0
**Elisabeth Mahase**[5]	1	Humanitarian	Gaza: "unprecedented" bombing of hospital leaves hundreds dead and injured	BMJ	18-Oct-23	News	UK	93.6	1	Neutral	1	0	1	0	0
**Tiago Correia**[6]	1	Opinon / Politic	Two urgent actions related to international health emergencies amid the escalating conflict in Gaza	International Journal of Health Planning and Management	18-Oct-23	Correspondence / Letter / Editorial	Portugal	1.9	3	pro-Israel	1	0	0	1	0
**Muhammad Abu Salmiya**[7]	1	Humanitarian	Urgent humanitarian call to save lives in Gaza	The Lancet	18-Oct-23	Correspondence / Letter / Editorial	Gaza / Palestine	98.4	1	pro-Gaza	1	0	1	0	0
**Gili Givaty**[8]	3	Humanitarian	Insights from the nearest Israeli hospital to the Gaza Strip	The Lancet	18-Oct-23	Correspondence / Letter / Editorial	Israel	98.4	1	pro-Israel	0	0	0	0	0
**Abdallah Abudayya**[9]	14	Humanitarian	An urgent call to save and protect lives of vulnerable populations in the Gaza Strip	The Lancet	21-Oct-23	Correspondence / Letter / Editorial	Norway	98.4	1	pro-Gaza	1	0	0	0	0

Author	Author	Topic	Title Journal	Publication Publication first author	Country of IF Journal Q	Journal In	Position In affiliation	Gaza *(referring*	Israel 7	Occupation	October Title	Genocide Title	number *to the Israeli occupation of Palestine)*	Date Citation	Type
**Akihiro Seita**[10]	2	Humanitarian	Gaza is facing a humanitarian catastrophe	The Lancet	23-Oct-23	Correspondence / Letter / Editorial	Jordan	98.4	1	pro-Gaza	1	0	0	0	0
**Owen Dyer**[11]	1	Humanitarian	Gaza hospitals are within hours of losing power as fuel embargo continues, say doctors and agencies	BMJ	24-Oct-23	News	Canada	93.6	1	pro-Gaza	1	0	0	0	0
**Paulo Buss**[12]	3	Humanitarian	End the violence and help the victims in Gaza	The Lancet	24-Oct-23	Correspondence / Letter / Editorial	Brazil	98.4	1	pro-Gaza	1	0	0	0	0
**Mohamed Reyad Zughbur**[13]	1	Humanitarian	Protect civilians' lives and health care in Gaza	The Lancet	27-Oct-23	Correspondence / Letter / Editorial	Gaza / Palestine	98.4	1	pro-Gaza	1	0	0	0	0
**Michele Chabin**[14]	1	Humanitarian	Israel-Hamas war shakes scientific community	Science & Society	27-Oct-23	News	Israel	0	NR	pro-Israel	0	1	0	1	0
**Samah Jabr**[15]	2	Humanitarian	Palestine meeting Gaza's mental health crisis	The Lancet	28-Oct-23	Correspondence / Letter / Editorial	Gaza / Palestine	98.4	1	pro-Gaza	1	0	0	0	0
**Richard Horton**[16]	1	Humanitarian	Offline: Israel–Gaza—what comes next?	The Lancet	28-Oct-23	Correspondence / Letter / Editorial	UK	98.4	1	pro-Israel	0	0	0	0	0
**Masoud LatifiPour**[17]	2	Humanitarian	Call for Emergency Aid for Gaza Strip Hospitals and Clinics	Trauma Monthly	29-Oct-23	Correspondence / Letter / Editorial	Iran	0.2	4	pro-Gaza	1	0	0	0	0
**Hasan Goodarzi**[18]	3	Humanitarian	War, Forced Displacement, and Lack of Healthcare in the Gaza Strip	Trauma Monthly	30-Oct-23	Correspondence / Letter / Editorial	Iran	0.2	4	pro-Gaza	1	0	0	0	0
**Shahin Akhondzadeh**[19]	1	Opinon / Politic	What is the Tolerance Limit of the Human Society in Face of the Killing of Innocent and Defenseless People of Gaza? Stop the Killing of Children and Infants in Gaza	Arch Iran Med	01-Nov-23	Correspondence / Letter / Editorial	Iran	1.0	3	pro-Gaza	1	0	0	0	1
**Shelley A Sternberg**[20]	2	Opinon / Politic	The conflict in Gaza: a view from Israel	The Lancet	01-Nov-23	Correspondence / Letter / Editorial	Israel	98.4	1	pro-Israel	0	0	0	0	0
**Elisabeth Mahase**[21]	1	Humanitarian	Gaza: UN warns that "nowhere is safe " for patients amid "relentless bombing campaign"	BMJ	02-Nov-23	News	UK	93.6	1	pro-Gaza	1	0	1	0	0

Author	Author	Topic	Title	Journal	Publication	Publication first author	Country of IF	Journal Q	Journal In	Position In affiliation	Gaza *(referring*	Israel 7	Occupation	October Title	Genocide number Title *to the Israeli occupation of Palestine)* Date Citation Type
**Ru'a Rimawi**[22]	2	Humanitarian	Gaza's broken health-care system	Nature	02-Nov-23	Correspondence / Letter / Editorial	USA	50.5	1	pro-Gaza	0	0	0	0	0
**Nicola Jones**[23]	1	Opinon / Politic	'I'm a powder keg': ousted eLife editor on being fired in wake of Israel–Hamas remarks	Nature	02-Nov-23	News	Canada	50.5	1	pro-Gaza	0	1	0	0	0
**Shahin Akhondzadeh**[24]	1	Opinon / Politic	Missile Attacks on Al-Ahli Hospital in Gaza and Silence of Reference International Authorities	Journal of Iranian Medical Council	03-Nov-23	Correspondence / Letter / Editorial	Iran	0	4	pro-Gaza	1	0	0	0	0
**Richard Horton**[25]	1	Opinon / Politic	Offline: Gaza's children a responsibility to protect	The Lancet	04-Nov-23	Correspondence / Letter / Editorial	UK	98.4	1	pro-Israel	1	0	1	0	0
**Abdulqadir J. Nashwan**[26]	1	Humanitarian	A Double Battle: Fighting Cancer in the Shadows of Conflict in Gaza	Cureus Journal of Medical Science	06-Nov-23	Correspondence / Letter / Editorial	Qatar	1.0	3	pro-Gaza	1	0	0	0	0
**Talha Burki**[27]	1	Humanitarian	Last cancer hospital in Gaza	The Lancet	09-Nov-23	News	UK	98.4	1	pro-Gaza	1	0	0	0	0
**Hussam Hussein**[28]	1	Humanitarian	Stop violation of international water laws in Gaza	Nature	09-Nov-23	Correspondence / Letter / Editorial	Jordan	50.5	1	pro-Gaza	1	0	0	0	0
**Adi Torfstein**[29]	3	Science	Israel: when reality meets academia	Nature	09-Nov-23	Correspondence / Letter / Editorial	Israel	50.5	1	pro-Israel	0	1	0	1	0
**Arif Khwaja**[30]	5	Opinon / Politic	Gaza: A plea to reclaim our collective humanity	The Lancet	10-Nov-23	Correspondence / Letter / Editorial	UK	98.4	1	Neutral	1	0	0	1	1
**Sheraz Yaqub**[31]	5	Opinon / Politic	Israel and Gaza: the killing of civilians must stop	The Lancet	10-Nov-23	Correspondence / Letter / Editorial	Norway	98.4	1	pro-Israel	1	0	0	1	0
**James Smith**[32]	24	Opinon / Politic	Palestine and Israel: for an end to violence and the pursuit of justice	The Lancet	10-Nov-23	Correspondence / Letter / Editorial	UK	98.4	1	pro-Gaza	0	0	0	0	0
**Sharmila Devi**[33]	1	Humanitarian	Health care in Gaza continues to worsen under conflict	The Lancet	11-Nov-23	Correspondence / Letter / Editorial	UK	98.4	1	pro-Gaza	1	0	0	1	0
**Luke Taylor**[34]	1	Opinon / Politic	Stop attacks on Gaza's hospitals, agencies plead, amid collapse of services	BMJ	13-Nov-23	News	UK	93.6	1	pro-Gaza	1	0	0	0	0

Author	Author	Topic	Title	Journal Publication	Publication first author Country of IF	Journal Q Journal In	Position In affiliation	Gaza (referring	Israel 7	Occupation	October Title	Genocide Title	number *to the Israeli occupation of Palestine)*	Date Citation	Type
**Shani Levany**[35]	3	Humanitarian	Calling for an immediate release of captive children in Gaza	The Lancet	14-Nov-23	Correspondence / Letter / Editorial	Israel	98.4	1	pro-Israel	1	0	0	1	0
**Sally Howard**[36]	1	Opinon / Politic	Doctors call on GMC to publish guidance on social media posts on the Israel Hamas war	BMJ	15-Nov-23	News	UK	93.6	1	pro-Israel	0	0	0	0	0
**Foad Aodi**[37]	1	Humanitarian	Israel-Hamas conflict: a call for peace in support of the Gaza population, where women and children are paying the highest price	Journal of Sex- and Gender-Specific Medicine	16-Nov-23	Correspondence / Letter / Editorial	Italy	0	1	pro-Israel	0	0	0	0	0
**Ora Peleg**[38]	2	Humanitarian	Internally displaced people amidst war: the Israeli narrative	The Lancet	16-Nov-23	Correspondence / Letter / Editorial	Israel	98.4	1	pro-Israel	0	1	0	1	0
**Reporters**[39]		Humanitarian	The Israel–Hamas conflict: voices from scientists on the front lines	Nature	16-Nov-23	News	USA	50.5	1	pro-Israel	0	0	0	1	0
**Duha Shellah**[40]	1	Humanitarian	How much suffering is enough? A look at what is happening in Gaza	The Lancet	17-Nov-23	Correspondence / Letter / Editorial	Gaza / Palestine	98.4	1	pro-Gaza	1	0	1	0	0
**Espen Bjertness**[41]	6	Humanitarian	Save the remaining people of Gaza—save the children	The Lancet	17-Nov-23	Correspondence / Letter / Editorial	Norway	98.4	1	pro-Gaza	1	0	0	0	1
**N.A. Uvais**[42]	2	Psychological	Jaspersian Principles for Achieving Lasting Peace in the PalestineIsrael Conflict	Asian Journal Of Psychiatry	20-Nov-23	Correspondence / Letter / Editorial	India	3.8	1	Neutral	0	0	4	0	0
**Maliheh Kadivar**[43]	1	Opinon / Politic	In Search of a Safe Place in Gaza for Children Stop Violence Against Children	Journal of Iranian Medical Council	20-Nov-23	Correspondence / Letter / Editorial	Iran	0	4	pro-Gaza	1	0	0	0	0
**Susan J Rees**[44]	2	Psychological	Invisible wounds of the Israel–Gaza war in Australia	Medical Journal Of Australia	20-Nov-23	Correspondence / Letter / Editorial	Australia	6.7	1	Neutral	0	0	1	0	0
**Owen Dyer**[45]	1	Humanitarian	More Gaza hospitals come under fire as Israeli forces search al-Shifa	BMJ	21-Nov-23	News	UK	93.6	1	pro-Gaza	0	0	0	0	0
**Fatemeh Beiraghda**[46]	5	Opinon / Politic	Health Crisis in Gaza: The Urgent Need for International Action	Iranian Journal of Public Health	21-Nov-23	Correspondence / Letter / Editorial	Iran	1.3	4	pro-Gaza	1	0	1	0	

Author	Author	Topic	Title Journal	Publication Publication first author	Country of IF Journal Q	Journal In	Position In affiliation	Gaza *(referring*	Israel 7	Occupation	October Title	Title	Genocide number *to the Israeli occupation of Palestine)*	Date Citation	Type
**Nitsa Nacasch**[47]	5	Psychological	Prevention of Post-traumatic stress disorder by Early Psychological Interventions Following the October 7th Massacre in Israel	Israel Medical Association Journal	25-Nov-23	Original / Review	Israel	1.8	2	pro-Israel	0	1	0	1	0
**Krystel Moussally**[48]	4	Science	Antimicrobial resistance in the ongoing Gaza war: a silent threat	The Lancet	25-Nov-23	Correspondence / Letter / Editorial	Israel	98.4	1	Neutral	1	0	0	0	0
**Zeina Jamaluddine**[49]	3	Humanitarian	Excess mortality in Gaza: Oct 7–26, 2023	The Lancet	27-Nov-23	Correspondence / Letter / Editorial	Japan	98.4	1	pro-Gaza	1	0	0	0	0
**Muhammad Abu Salmiya**[50]	1	Humanitarian	Gaza hospitals military siege and bombings	The Lancet	27-Nov-23	Correspondence / Letter / Editorial	Gaza / Palestine	98.4	1	pro-Gaza	1	0	0	0	0
**Zohar Elyoseph**[51]	7	Psychological	Mental health volunteers after the Oct 7 Gaza border crisis in Israel: silent warriors	The Lancet	29-Nov-23	Correspondence / Letter / Editorial	Israel	98.4	1	pro-Israel	0	1	0	1	0
**Karzan Qurbani**[52]	4	Psychological	Urgent attention needed for the mental health of people in Gaza: A call for global action	Asian Journal Of Psychiatry	30-Nov-23	Correspondence / Letter / Editorial	Iraq	3.8	1	Neutral	1	0	0	1	0
**John Launer**[53]	1	Opinon / Politic	John Launer: Israel and Gaza recognising shared human values	BMJ	30-Nov-23	Correspondence / Letter / Editorial	UK	93.6	1	pro-Israel	0	0	0	0	0
**Ayesha Musa**[54]	5	Humanitarian	Gaza, 9 years on a humanitarian catastrophe.	The Lancet	30-Nov-23	Correspondence / Letter / Editorial	UK	98.4	1	pro-Gaza	1	0	0	0	0
**Maximilian P. Nerlander**[55]	4	Science	Strengthening Preparedness Against Terror: A Bystander Training Concept by Israel's Emergency Medical Services.	American Surgeon	30-Nov-23	Original / Review	Israel	1.0	3	pro-Israel	0	1	0	1	0
**Helmi Ben Saad**[56]	7	Humanitarian	Gaza Genocide Breaking the silence.	La Tunisie Medicale	30-Nov-23	Correspondence / Letter / Editorial	Tunisia	0	4	pro-Gaza	1	0	0	0	1
**Ahmed Al-Mandhari**[57]	6	Humanitarian	Gaza disaster: we need a permanent ceasefire, now!	Eastern Mediterranean Health Journal	01-Dec-23	Correspondence / Letter / Editorial	WHO	1.7	3	pro-Gaza	1	0	0	1	0
**Moshe Salai**[58]															

Author	Author	Topic	Title Journal	Publication Publication first author	Country of IF Journal Q	Journal In	Position In affiliation	Gaza *(referring*	Israel 7	Occupation	October Title	Title	Genocide number *to the Israeli occupation of Palestine)*	Date Citation	Type
	10	Opinon / Politic	Desecration by Hamas of the Holy Ten Commandments Embedded in Medical Education during the Iron Swords War in Gaza.	Israel Medical Association Journal	01-Dec-23	Original / Review	Israel	1.8	2	pro-Israel	0	0	0	1	0
**Gabriel Levin**[59]	3	Opinon / Politic	Analysis of the scientific publications on the Gaza-Israeli conflict.	Israel Medical Association Journal	01-Dec-23	Original / Review	Canada	1.8	2	pro-Israel	0	0	0	1	0
**Chen Kugel**[60]	5	Science	Forensic Frontiers Navigating Complex Challenges of a Largescale Invasion by Armed Hamas Terrorists in Southern Israel.	Israel Medical Association Journal	01-Dec-23	Original / Review	Israel	1.8	2	pro-Israel	0	0	0	1	0
**Hussam Mahmoud**[61]	2	Humanitarian	State of the health-care system in Gaza during the Israel–Hamas war.	The Lancet	01-Dec-23	Correspondence / Letter / Editorial	USA	98.4	1	pro-Gaza	0	0	0	0	0
**S Soni**[62] **S Mahomed**[63]	1	Opinon / Politic	Gaza and international law: The global obligation to protect life and health.	South African Journal of Bioethics and Law	01-Dec-23	Correspondence / Letter / Editorial	South Africa	0.5	4	pro-Gaza	1	0	1	1	2
	1	Opinon / Politic	When sanctuaries of humanity turn into corridors of horror: The destruction of healthcare in Gaza.	South African Journal of Bioethics and Law	01-Dec-23	Correspondence / Letter / Editorial	South Africa	0.5	4	pro-Gaza	1	0	3	0	1
**Marcos Arana-Cedeño**[64]	1	Opinon / Politic	Hospitals, health and death in Gaza.	Social Medicine	01-Dec-23	Correspondence / Letter / Editorial	Mexico	0	4	pro-Gaza	1	0	1	0	4
**Elisabeth Mahase**[65]	1	Humanitarian	Conditions in Gaza are ripe for "epidemics and public health disaster," UN warns.	BMJ	06-Dec-23	News	UK	93.6	1	pro-Gaza	1	0	1	0	0
**Sirwan K. Ahmed**[66]	1	Humanitarian	Addressing the Effects of War on Gaza's Healthcare System.	Cureus Journal of Medical Science	06-Dec-23	Correspondence / Letter / Editorial	Iraq	1.0	3	pro-Gaza	1	0	0	0	0
**Benjamin Q Huynh**[67]	3	Science	No evidence of inflated mortality reporting from the Gaza ministry of health.	The Lancet	06-Dec-23	Correspondence / Letter / Editorial	USA	98.4	1	Neutral	1	0	0	0	0
**Soraida HusseinSabbah**[68]		Humanitarian	Pregnant women in Gaza face perilous conditions as maternity services and infrastructure crumble	BMJ	07-Dec-23	Correspondence / Letter / Editorial	Spain	93.6	1	pro-Gaza	1	0	1	0	0

Author	Author	Topic	Title	Journal	Publication	Publication first author	Country of IF	Journal Q	Journal In	Position In affiliation	Gaza *(referring*	Israel 7	Occupation	October Title	Genocide number Title *to the Israeli occupation of Palestine)* Date Citation Type
**Amitava Banerjee**[69]	1	Humanitarian	The conflict in Gaza—are data ever enough?	The Lancet	07-Dec-23	Correspondence / Letter / Editorial	UK	98.4	1	pro-Gaza	1	0	0	0	0
**Salam (pseudonym)**[70]	1	Humanitarian	There is no way to leave Gaza.	The Lancet	08-Dec-23	Correspondence / Letter / Editorial	Gaza / Palestine	98.4	1	pro-Gaza	1	0	0	0	0
**Richard Horton**[71]	1	Opinon / Politic	Offline: "My brothers and sisters in Israel and Palestine"	The Lancet	09-Dec-23	Correspondence / Letter / Editorial	USA	98.4	1	pro-Israel	0	0	0	0	1
**WHO**[72]		Humanitarian	WHO's Executive Board adopts resolution on access for life-saving aid into Gaza and respect for laws of war	WHO	10-Dec-23	News	WHO	-	-	pro-Gaza	1	0	0	0	0
**John Zarocostas**[73]	1	Humanitarian	WHO Executive Board calls for action in Gaza	The Lancet	16-Dec-23	News	WHO	98.4	1	pro-Gaza	1	0	0	0	0
**Alison Shepherd**[74]	1	Humanitarian	Help women in Gaza and across the globe	BMJ	20-Dec-23	News	UK	93.6	1	pro-Gaza	1	0	0	0	0
**A. Mark Clarfield**[75]	1	Science	Older persons, new challenges: Caring for vulnerable persons during wartime in Israel	Journal of The American Geriatrics Society	20-Dec-23	Correspondence / Letter / Editorial	Israel	4.p3	1	pro-Israel	0	1	0	1	0
**Jerome Teitel**[76]	1	Opinon / Politic	Israel and Gaza: a quick end to a tragic but necessary war	The Lancet	20-Dec-23	Correspondence / Letter / Editorial	Canada	98.4	1	pro-Israel	0	0	0	0	1
**Ames Dhai**[77]	1	Humanitarian	The situation in Gaza – will cruelty and hatred triumph?	South African Journal of Bioethics and Law	20-Dec-23	Correspondence / Letter / Editorial	South Africa	0.5	4	pro-Gaza	1	0	0	1	7
**Gene Feder** [78]	4	Humanitarian	Responding to the war in Israel and Palestine.	British Journal of General Practice	22-Dec-23	Correspondence / Letter / Editorial	UK	5.3	1	pro-Israel	0	0	1	0	0
**Fahdah Alokaily**[79]	1	Humanitarian	War and Health Crisis in Gaza.	Saudi Medical Journal	01-Jan-24	Correspondence / Letter / Editorial	Saudi Arabia	1.7	2	pro-Gaza	1	0	0	0	0
**Abirami Muthumani**[80]	1	Humanitarian	Safeguarding children through pediatric surgical care in war and humanitarian settings: a call to action for pediatric patients in Gaza.	BMJ	02-Jan-24	Correspondence / Letter / Editorial	USA	93.6	1	pro-Gaza	1	0	0	0	0

Author	Author	Topic	Title Journal	Publication Publication first author	Country of IF Journal Q	Journal In	Position In affiliation	Gaza *(referring*	Israel 7	Occupation	October Title	Title	Genocide number *to the Israeli occupation of Palestine)*	Date Citation	Type
**Shlomi Codish[81]**	6	Humanitarian	October 7th 2023 attacks in Israel: frontline experience of a single tertiary center.	Intensive Care Medicine	03-Jan-24	Correspondence / Letter / Editorial	Israel	27.1	1	pro-Israel	0	1	0	1	0
**Yossi LeviBelz[82]**	4	Psychological	PTSD, depression, and anxiety after the October 7, 2023 attack in Israel: a nationwide prospective study.	E Clinical Medicine	05-Jan-24	Original / Review	Israel	9.6	1	pro-Israel	0	1	0	1	0
**Elisabeth Mahase[83]**	1	Humanitarian	Gaza: who describes "sickening scene" in hospital as Israeli military intensifies activity.	BMJ	09-Jan-24	News	UK	93.6	1	pro-Gaza	0	0	1	0	0
**Shatha Elnakib[84]**	5	Humanitarian	Pregnant women in Gaza require urgent protection.	The Lancet	11-Jan-24	Correspondence / Letter / Editorial	USA	98.4	1	pro-Gaza	1	0	0	0	0
**Joel B Zivot[85]**	5	Opinon / Politic	Gaza and the complexity and context of suffering.	The Lancet	11-Jan-24	Correspondence / Letter / Editorial	USA	98.4	1	pro-Israel	1	0	0	0	0
**Mennatt Allah Hassan Attia[86]**	1	Humanitarian	A call for the initiation of the forensic humanitarian action in Gaza.	Egyptian Journal Of Forensic Sciences	13-Jan-24	Correspondence / Letter / Editorial	Egypt	1.3	3	pro-Gaza	0	0	1	0	0
**Kamran Bagheri Lankarani[87]**	1	Humanitarian	The War of Gaza and International Laws	Shiraz Electronic Medical Journal	15-Jan-24	Correspondence / Letter / Editorial	Iran	0	NR	pro-Gaza	1	0	1	0	0
**Luke Taylor[88]**	1	Humanitarian	Allow more aid into Gaza or risk famine, warns WHO	BMJ	16-Jan-24	News	UK	93.6	1	pro-Gaza	1	0	0	1	0
**LawrenceO. Gostin[89]**	2	Opinon / Politic	Wars in Gaza and Beyond Why Protecting the Sacredness of Health Matters	JAMA	16-Jan-24	Correspondence / Letter / Editorial	USA	63.1	1	pro-Gaza	1	0	2	1	1
**Rachel Coghlan[90]**	3	Humanitarian	Morphine for Gaza? Limits of care during genocidal violence	The Lancet	16-Jan-24	Correspondence / Letter / Editorial	Australia	98.4	1	pro-Gaza	1	0	1	0	1
**Luis Eugenio Souza[91]**	1	Opinon / Politic	Wars are never necessary: Gaza is the best example	The Lancet	16-Jan-24	Correspondence / Letter / Editorial	Switzerland	98.4	1	pro-Gaza	1	0	0	0	0
**Thalia Arawi[92]**	1	Humanitarian	War on healthcare services in Gaza	Indian Journal of Medical Ethics	17-Jan-24	Correspondence / Letter / Editorial	Lebanon	0	3	pro-Gaza	1	0	5	0	1
**Emmanuel**	4	Humanitarian	Gaza ceasefire and prepare for a surge in infectious diseases.	Postgraduate Medical Journal	20-Jan-24	Correspondence / Letter /	UAE	3.6	1	pro-Gaza	1	0	0	0	0

Author	Author	Topic	Title	Journal	Publication / Publication first author	Country of IF / Journal Q	Journal In	Position In affiliation	Gaza *(referring*	Israel 7	Occupation	October Title	Genocide Title	number *to the Israeli occupation of Palestine)*	Date Citation	Type
**Nsutebu**[93]							Editorial									
**Duha Shellah**[94]	1	Humanitarian	War on Gaza: the impossible duty to care for the critically ill.	Intensive Care Medicine	22-Jan-24		Correspondence / Letter / Editorial	Gaza / Palestine	27.1	1	pro-Gaza	1	0	1	0	0
**Elisabeth Mahase**[95]	1	Humanitarian	Gaza-Israel conflict: hundreds of medics are killed or arrested after intense attacks on healthcare facilities.	BMJ	25-Jan-24		News	UK	93.6	1	pro-Gaza	0	0	0	0	0
**Mun-Keat Looi**[96]	1	Humanitarian	The BMJ Appeal 2023-24: Gaza's mothers and babies need your help.	BMJ	25-Jan-24		Correspondence / Letter / Editorial	UK	93.6	1	pro-Gaza	1	0	0	0	0
**Evan Avraham Alpert**[97]	5	Science	Secondary Ambulance Transfers During the Mass-Casualty Terrorist Attack in Israel on October 7, 2023	Prehospital and Disaster Medicine	25-Jan-24		Original / Review	Israel	2.1	2	pro-Israel	0	1	0	1	0
**Ofer Almog**[98]	6	Science	Deploying whole blood to the battlefield—The Israel Defense Forces Medical Corps initial experience during the 2023 war	Transfusion	28-Jan-24		Original / Review	Israel	0.2	4	pro-Israel	0	1	0	0	0
**Amira Mohamed Taha**[99]	4	Science	Controlling the alarming rise in infectious diseases among children younger than 5 years in Gaza during the war	The Lancet	30-Jan-24		Correspondence / Letter / Editorial	Egypt	98.4	1	pro-Gaza	1	0	1	0	0
**Philip Greenland**[100]	3	Opinon / Politic	Importance of a broader view of the Hamas–Israel war	BMJ Public Health	01-Feb-24		Correspondence / Letter / Editorial	USA	3.5	1	pro-Israel	0	0	6	1	0
**Aryeh Lazara**[101]	3	Psychological	War-Time Stress and Sexual Well-Being in Israel	International Journal Of Sexual Health	03-Feb-24		Original / Review	Israel	2.2	1	pro-Israel	0	1	0	1	0
**Liat Ayalon**[102]	6	Psychological	On Intergenerational Conflict and Solidarity at Times of Terror and War in Israel: The Case of Late-Life Physical Vulnerabilities and Emotional Resilience	Am J Geriatr Psychiatry	05-Feb-24		Correspondence / Letter / Editorial	Israel	4.4	1	pro-Israel	0	1	0	0	0
**Helmi Ben Saad**[103]	1	Humanitarian	Urgent humanitarian appeal: Protecting the lives of women and children in the Gaza Strip (Palestine)	Journal of Public Health Research	05-Feb-24		Correspondence / Letter / Editorial	Tunisia	1.6	3	pro-Gaza	1	0	0	0	1

Author	Author	Topic	Title Journal	Publication Publication first author	Country of IF Journal Q	Journal In	Position In affiliation	Gaza *(referring*	Israel 7	Occupation	October Title	Genocide number Title	*to the Israeli occupation of Palestine)*	Date Citation	Type
**John E. Kearney**[104]	8	Opinon / Politic	Conflicts in Gaza and around the world create a perfect storm for infectious disease outbreaks	Plos Global Public Health	07-Feb-24	Correspondence / Letter / Editorial	USA	0	NR	pro-Gaza	1	0	0	0	0
**Amira Mohamed Taha**[105]	5	Humanitarian	Addressing the mental health crisis among children in Gaza.	The Lancet	09-Feb-24	Correspondence / Letter / Editorial	Egypt	98.4	1	pro-Gaza	1	0	0	0	0
**Danielle N Poole**[106]	7	Humanitarian	Damage to medical complexes in the Gaza Strip during the Israel–Hamas war: a geospatial analysis.	BMJ Global Health	11-Feb-24	Original / Review	USA	7.1	2	pro-Gaza	0	0	7	0	0
**Mohammed Qandil**[107]	1	Humanitarian	Gaza providing emergency care under fire.	Emergency Medicine Journal	11-Feb-24	Correspondence / Letter / Editorial	Gaza / Palestine	2.7	1	pro-Gaza	1	0	0	0	0
**Yamina Boukari**[108]	11	Humanitarian	Gaza, armed conflict and child health.	BMJ	13-Feb-24	Correspondence / Letter / Editorial	UK	93.6	1	pro-Gaza	1	0	0	1	0
**Ghada Al-Jadba**[109]	6	Humanitarian	UNRWA at the front lines: managing health care in Gaza during catastrophe.	The Lancet	13-Feb-24	Correspondence / Letter / Editorial	Gaza / Palestine	98.4	1	pro-Gaza	1	0	0	0	0
**Mishal S Khan**[110]	2	Humanitarian	Israel–Palestine: dehumanisation and silencing	The Lancet	16-Feb-24	Correspondence / Letter / Editorial	UK	98.4	1	pro-Gaza	0	0	0	0	1
**Elisabeth Mahase**[111]	1	Humanitarian	Gaza: doctors of the world office destroyed, as medics are forced to amputate without anaesthetic	BMJ	19-Feb-24	News	UK	93.6	1	pro-Gaza	1	0	0	0	0
**Mun Keat Looi**[112]	1	Humanitarian	Gaza: "no health system left" says MSF	BMJ	23-Feb-24	News	UK	93.6	1	pro-Gaza	1	0	0	0	0
**Oliver Razum**[113]	12	Opinon / Politic	Statement of the ASPHER Task Force on War and Public Health on the Conflict in Israel/Palestine	Public Health Reviews	23-Feb-24	Correspondence / Letter / Editorial	Germany	3.5	1	pro-Israel	0	0	0	1	1
**Zohar Lederman**[114]	3	Opinon / Politic	Making a case for appropriate and humane treatment of Hamas belligerents in Israel.	American Journal of Bioethics	24-Feb-24	Correspondence / Letter / Editorial	China	17	1	Neutral	0	1	0	1	0
**Hanan Balkhy**[115]	1	Humanitarian	As sickness, hunger, and disease spread, Gaza's health system faces increasing threats	BMJ	29-Feb-24	Correspondence / Letter / Editorial	WHO	93.6	1	pro-Gaza	1	0	1	0	0

Author	Author	Topic	Title	Journal	Publication Publication first author Country of IF Journal Q	Journal In	Position In affiliation	Gaza (referring	Israel 7	Occupation	October Title	Genocide Title	number *to the Israeli occupation of Palestine)*	Date Citation	Type
**Eli Jaffe**[116]	8	Science	Managing a Mega Mass Casualty Event by a Civilian Emergency Medical Services Agency: Lessons From the First Day of the 2023 Hamas-Israel War	International Journal Of Public Health	29-Feb-24	Original / Review	Israel	2.6	2	Neutral	0	0	0	1	0
**Joseph Medlovic**[117]	3	Opinon / Politic	A Statistical Approach to the High Mortality Rate of Israeli Citizens Held Hostage in Gaza	Israel Medical Association Journal	01-Mar-24	Original / Review	Israel	1.8	2	pro-Israel	0	0	0	1	0
**Somaya Albhaisi**[118]	1	Humanitarian	The impossible mission: to save and support science in Gaza	The Lancet	02-Mar-24	Correspondence / Letter / Editorial	USA	98.4	1	pro-Gaza	1	0	1	0	0
**Arielle Kaim**[119]	6	Science	A longitudinal study of societal resilience and its predictors during the IsraelGaza war	Appl Psychol Health WellBeing	04-Mar-24	Original / Review	Israel	3.8	1	pro-Israel	0	0	0	1	0
**Muna Abed Alah**[120]	1	Humanitarian	Echoes of conflict: the enduring mental health struggle of Gaza's healthcare workers	Conflict And Health	04-Mar-24	Correspondence / Letter / Editorial	Qatar	3.1	2	pro-Gaza	1	0	0	0	0
**Sheila M Bird**[121]	1	Science	Threat level in the Gaza Strip: fatalities per 1000 person-years	The Lancet	05-Mar-24	Correspondence / Letter / Editorial	UK	98.4	1	Neutral	1	0	0	0	0
**Safiyyah Abbas**[122]	2	Opinon / Politic	Australian medical leadership's silence on Gaza is a moral failure	The Lancet	05-Mar-24	Correspondence / Letter / Editorial	Australia	98.4	1	pro-Gaza	1	0	1	0	2
**Mohamed Adil Shah**[123] **Khoodoruth**	2	Science	From past famines to present crises: The epigenetic impact of maternal malnutrition on offspring health in Gaza	Asian Journal Of Psychiatry	11-Mar-24	Correspondence / Letter / Editorial	Qatar	3.8	1	pro-Gaza	1	0	0	0	0
**Yara Asi**[124]	10	Humanitarian	'Nowhere and no one is safe': spatial analysis of damage to critical civilian infrastructure in the Gaza Strip during the first phase of the Israeli military campaign.	Conflict And Health	11-Mar-24	Original / Review	USA	3.1	2	pro-Gaza	0	0	1	0	0
**Elisabeth Mahase**[125]	1	Humanitarian	Gaza: Children dying of starvation as UN food trucks turned away, say aid organisations	BMJ	12-Mar-24	News	UK	93.6	1	pro-Gaza	1	0	0	0	0
**Cameron**	5	Humanitarian	Women and girls in Gaza face increasingly dire physical and mental health challenges	BMJ	12-Mar-24	Correspondence / Letter / Editorial	USA	93.6	1	pro-Gaza	1	0	0	1	0

Author	Author	Topic	Title Journal	Publication Publication first author	Country of IF Journal Q	Journal In	Position In affiliation	Gaza (referring	Israel 7	Occupation	October Title	Title	Genocide number *to the Israeli occupation of Palestine)*	Date Citation	Type
**Sabet**[126]	2	Opinon / Politic	International Humanitarian Law in the Israeli-Gaza Conflict—Reply	JAMA	13-Mar-24	Correspondence / Letter / Editorial	USA	63.1	1	pro-Gaza	0	0	0	0	0
**Lawrence O. Gostin**[127]	1	Humanitarian	Gaza: BMA calls for urgent investigation into mistreatment of doctors	BMJ	14-Mar-24	News	UK	93.6	1	pro-Gaza	1	0	0	0	0
**Elisabeth Mahase**[128]	2	Science	A unique combination of horror and longing: Traumatic grief in post–October 7, 2023, Israel	Journal of Traumatic Stress	15-Mar-24	Correspondence / Letter / Editorial	Israel	2.4	2	pro-Israel	0	1	0	1	0
**Ilanit Hasson-Ohayon**[129]	24	Humanitarian	Gaza War: Too many citizens being killed.	British Journal of Surgery	16-Mar-24	Correspondence / Letter / Editorial	USA	8.6	1	pro-Gaza	1	0	0	0	1
**Sheraz Yaqub**[130]	3	Psychological	Factors associated with insomnia and fatigue symptoms during the outbreak of Oct.7th war on Gaza: A study from Jordan	Preventive Medicine Reports	16-Mar-24	Original / Review	Jordan	2.4	2	Neutral	1	0	0	0	0
**Omar Salem Gammoh**[131]	2	Science	Age differences in acute stress and PTSD symptoms during the 2023 Israel-Hamas war: Preliminary findings	Journal of Psychiatric Research	19-Mar-24	Original / Review	Israel	3.7	1	pro-Israel	0	0	0	0	0
**Amit Shrira**[132]	5	Science	Evaluating emergency response at a hospital near the Gaza border within 24 h of increased conflict	BMC Emergency Medicine	21-Mar-24	Original / Review	Israel	2.3	1	pro-Israel	1	0	0	1	0
**Hezi Levi**[133]	1	Humanitarian	Gaza: "Hospitals should never be battlegrounds," says WHO amid raids on al-Shifa hospital	BMJ	21-Mar-24	News	UK	93.6	1	pro-Gaza	1	0	1	0	0
**Elisabeth Mahase**[134]	1	Opinon / Politic	It is Not Too Late for Reconciliation Between Israel and Palestine, Even in the Darkest Hour	Bioethical Inquiry	22-Mar-24	Original / Review	Russia	0	NR	Neutral	0	0	2	0	0
**P. A. Komesaroff**[135]															
**Ken Menon**[136]	1	Humanitarian	Responding to the war in Israel and Palestine.	British Journal Of General Practice	27-Mar-24	Correspondence / Letter / Editorial	UK	5.3	1	Neutral	0	0	0	0	0
**Liat Ayalon**[137]	3	Psychological	Global Conflict and the Plight of Older Persons: Lessons From Israel.	Am J Geriatr Psychiatry	01-Apr-24	Correspondence / Letter / Editorial	Israel	4.4	1	pro-Israel	0	1	0	1	0

Author	Author	Topic	Title	Journal	Publication	Publication first author	Country of IF	Journal Q	Journal In	Position In affiliation	Gaza (referring	Israel 7	Occupation October Title	Genocide number Title *to the Israeli occupation of Palestine)*	Date Citation Type
**Rael D Strous**[138]	2	Psychological	The ethics of psychiatric management in times of disaster and war: experiences from Israel after the Oct 7 attack.	The Lancet	01-Apr-24	Correspondence / Letter / Editorial	Israel	98.4	1	pro-Israel	0	1	0	1	0
**Fatima Hassan**[139]	3	Opinon / Politic	The global health community must call for an immediate ceasefire and unrestricted humanitarian aid in Gaza.	BMJ	02-Apr-24	Correspondence / Letter / Editorial	South Africa	93.6	1	pro-Gaza	1	0	0	0	0
**Abhi C Lohana**[140]	5	Science	The Silent Victims: How the Israel-Palestine War Impacts the Management of Chronic Kidney Disease and End-Stage Renal Disease Patients.	Cureus Journal of Medical Science	03-Apr-24	Correspondence / Letter / Editorial	USA	1.0	3	Neutral	0	0	0	0	0
**Liat Helpman**[141]	5	Psychological	Creating new life while lives are lost: birth in the face of war in Israel after the October 7 attacks.	Journal of Reproductive And Infant Psychology	03-Apr-24	Correspondence / Letter / Editorial	Israel	2.1	2	pro-Israel	0	1	0	1	0
**Elisabeth Mahase**[142]	1	Opinon / Politic	Gaza: Muslim doctors in UK feel censored and targeted for expressing concern over humanitarian crisis.	BMJ	09-Apr-24	News	UK	93.6	1	pro-Gaza	1	0	0	0	0
**Eivind Engebretsen**[143]	2	Opinon / Politic	The Rhetoric of Decolonizing Global Health Fails to Address the Reality of Settler Colonialism: Gaza as a Case in Point	International Journal of Health Policy and Management	09-Apr-24	Correspondence / Letter / Editorial	Norway	3.1	1	pro-Gaza	1	0	2	0	0
**Steven Roth**[144]	5	Opinon / Politic	Hypocrisy of moral imperatives in the Israel–Hamas war	The Lancet	09-Apr-24	Correspondence / Letter / Editorial	USA	98.4	1	pro-Israel	0	0	0	1	1
**Andrea Moscatelli**[145]	5	Humanitarian	Medical evacuation challenges of children from Gaza	The Lancet	09-Apr-24	Correspondence / Letter / Editorial	Italy	98.4	1	pro-Gaza	1	0	0	0	0
**Linda Young Landesman**[146]	3	Opinon / Politic	Peace building Through Cooperation in Health Care and Public Health Between Israel and Palestine	Journal of Public Health Management and Practice	10-Apr-24	Correspondence / Letter / Editorial	USA	2.2	2	pro-Israel	0	0	0	0	0
**Yossi LeviBelz**[147]	5	Psychological	The moderating role of belongingness in the contribution of depression to suicide ideation following the October 7, 2023,	Journal of Affective Disorders	12-Apr-24	Original / Review	Israel	4.9	1	pro-Israel	0	1	0	1	0

Author	Author number	Topic	Title	Journal	Publication Date	Publication Type	Country of first author affiliation	Journal IF	Journal Q	Position	Gaza In Title	Israel In Title	Occupation (referring to the Israeli occupation of Palestine)	October 7 Title	Genocide Citation
			terrorist attack in Israel: A nationwide prospective study.												
**Daniel Feingold**[148]	3	Psychological	PTSD, distress and substance use in the aftermath of October 7th, 2023, terror attacks in Southern Israel	Journal of Psychiatric Research	13-Apr-24	Original / Review	Israel	3.7	1	pro-Israel	0	1	0	1	0
**Leora Brazg Ferro**[149]	6	Psychological	Radiation Therapy Delivery During the 2023 Israel-Hamas War: Trust Prevails Over Fear	Advances in Radiation Oncology	16-Apr-24	Original / Review	Israel	2.2	2	pro-Israel	0	0	0	1	0
**Sherri N. Sheinfeld Gorin**[150]	1	Humanitarian	International Humanitarian Law in the Israeli-Gaza Conflict	JAMA	16-Apr-24	Correspondence / Letter / Editorial	USA	63.1	1	pro-Israel	0	0	0	1	0
**Nikki R. Adler**[151]	3	Opinon / Politic	International Humanitarian Law in the Israeli-Gaza Conflict	JAMA	16-Apr-24	Correspondence / Letter / Editorial	Australia	63.1	1	pro-Israel	0	0	0	1	0
**Elihu D. Richter**[152]	3	Opinon / Politic	International Humanitarian Law in the Israeli-Gaza Conflict	JAMA	16-Apr-24	Correspondence / Letter / Editorial	Israel	63.1	1	pro-Israel	0	0	0	1	5
**Charles G. Kels**[153]	1	Opinon / Politic	International Humanitarian Law in the Israeli-Gaza conflict	JAMA	16-Apr-24	Correspondence / Letter / Editorial	USA	63.1	1	pro-Israel	0	0	0	0	0
**Omar Gammoh**[154]	2	Psychological	The prevalence and correlates of PTSD, insomnia, and fatigue among people with epilepsy during Oct.7th war on Gaza: A study from Jordan	Epilepsy & Behavior	17-Apr-24	Original / Review	Jordan	2.3	2	Neutral	1	0	1	0	0
**Samer Abuzerr**[155]	5	Humanitarian	The silent crisis: effect of malnutrition and dehydration on children in Gaza during the war	Frontiers Nutrition	22-Apr-24	Correspondence / Letter / Editorial	Gaza / Palestine	4.0	2	pro-Gaza	1	0	0	0	0
**Abdullah Al-Jamal**[156]	1	Psychological	Gaza	The Lancet	22-Apr-24	Correspondence / Letter / Editorial	UK	98.4	1	pro-Gaza	1	0	0	0	0
**Andrés Gautier Hirsch**[157]	1	Opinon / Politic	Peace between Israel and Palestine?	Torture	22-Apr-24	Correspondence / Letter / Editorial	Bolivia	0	3	pro-Gaza	0	0	0	1	0

Author	Author	Topic	Title	Journal	Publication Publication first author Country of IF	Journal Q Journal In	Position In affiliation	Gaza *(referring*	Israel 7	Occupation	October Title	Title	Genocide number *to the Israeli occupation of Palestine)*	Date Citation	Type
**Alex de Waal**[158]	1	Humanitarian	Famine in Gaza: an example of the global humanitarian crisis	American Journal of Clinical Nutrition	23-Apr-24	Correspondence / Letter / Editorial	USA	6.5	1	pro-Gaza	1	0	0	0	2
**Ora Paltiel**[159]	18	Humanitarian	Children on the Gaza-Israel Border: Victims of War	Public Health Reviews	24-Apr-24	Correspondence / Letter / Editorial	Israel	3.5	1	pro-Israel	0	0	0	1	0
**Nourit Houminer**[160] **Klepar**	3	Psychological	Emotional Eating among College Students in Israel: A Study during Times of War	Foods (MDPI)	27-Apr-24	Original / Review	Israel	4.7	1	pro-Israel	0	1	0	0	0
**Ruth Maytles**[161]	2	Psychological	Caring for Internally Displaced Older Adult Israelis during the 2023 Israel-Hamas War	Am J Geriatr Psychiatry	01-May-24	Correspondence / Letter / Editorial	Israel	4.4	1	pro-Israel	0	0	0	1	0
**Oliver Razum**[162]	5	Science	Gaza Ceasefire: Improve WASH, Promote Cooperation	International Journal Of Public Health	01-May-24	Correspondence / Letter / Editorial	Germany	2.6	2	Neutral	1	0	0	0	0
**Yara Ashour**[163]	3	Humanitarian	Patients in Gaza with chronic conditions need urgent interventions	The Lancet	01-May-24	Correspondence / Letter / Editorial	Gaza / Palestine	98.4	1	pro-Gaza	1	0	0	0	0
**Samar Abuzerr**[164]	2	Humanitarian	Amplifying the voices of women in the Gaza Strip	The Lancet	01-May-24	Correspondence / Letter / Editorial	Gaza / Palestine	98.4	1	pro-Gaza	1	0	0	0	0
**Shani Pitcho**[165]	1	Psychological	The stuff that nightmares are made of: Israeli dreams in times of the Israel-Hamas war	Death Studies	06-May-24	Original / Review	Israel	2.1	2	pro-Israel	0	0	0	1	0
**Sameer Sah**[166]	2	Humanitarian	Israel is using starvation as a weapon of war in Gaza	BMJ	07-May-24	Correspondence / Letter / Editorial	UK	93.6	1	pro-Gaza	0	0	0	0	0
**Arianne Shahvisi**[167]	1	Humanitarian	The ethical is political: Israel's production of health scarcity in Gaza	Journal of Medical Ethics	09-May-24	Correspondence / Letter / Editorial	UK	3.3	1	pro-Gaza	0	0	4	0	2
**Bram Wispelwey**[168]	6	Opinon / Politic	Civilian mortality and damage to medical facilities in Gaza	BMJ Global Health	16-May-24	Correspondence / Letter / Editorial	USA	7.1	2	pro-Gaza	1	0	1	0	4
**Genevieve Lloy**[169]	1	Opinon / Politic	Seeing Gaza: Objectivity and Emotion	Bioethical Inquiry	22-May-24	Correspondence / Letter / Editorial	Australia	0	NR	pro-Gaza	1	0	0	0	4

Author	Author	Topic	Title Journal	Publication Publication first author	Country of IF	Journal Q Journal In	Position In affiliation	Gaza *(referring*	Israel 7	Occupation	October Title	Genocide number Title	*to the Israeli occupation of Palestine)*	Date Citation	Type
**Dua'a AlMaghaireh**[170]	7	Psychological	Acute Stress Disorders Among Jordanian Adolescents After Watching Gaza News Footage on Social Media	Journal of Multidisciplinary Healthcare	22-May-24	Original / Review	Jordan	2.7	2	Neutral	1	0	0	0	0
**Ronen Segev**[171]	3	Humanitarian	Nurses under fire: Insights from testimonies of community nurses and midwives in nonhospital settings in the southern Israel conflict zone	Research in Nursing and Health	23-May-24	Original / Review	Israel	2.1	2	pro-Israel	0	1	0	1	0
**Pooja Yerramilli**[172]	1	Humanitarian	Refeeding in crisis settings: Implications on health care needs in Gaza	Plos Global Public Health	24-May-24	Correspondence / Letter / Editorial	USA	0	NR	pro-Gaza	1	0	0	0	0
**Muhammad Abu Salmiya**[173]	1	Humanitarian	Stop the Gaza genocide immediately	The Lancet	25-May-24	Correspondence / Letter / Editorial	Gaza / Palestine	98.4	1	pro-Gaza	1	0	0	1	1
**Inbar Levkovich**[174]	2	Psychological	"I Felt a Sense of Mission during Moments of Crisis": Mental Health Professionals' Perspectives on Their Initial Treatment of Evacuees during the Israel–Ham	Healthcare	27-May-24	Original / Review	Israel	2.4	2	pro-Israel	0	0	0	0	0
**Karl Blanchet**[175]	11	Humanitarian	Rebuilding the health sector in Gaza: alternative humanitarian voices	Conflict And Health	31-May-24	Correspondence / Letter / Editorial	Switzerland	3.1	2	pro-Gaza	1	0	0	0	0
**Feten Fekih-Romdhane**[176]	9	Psychological	Mediating effect of depression and acute stress between exposure to Israel-Gaza war media coverage and insomnia: a multinational study from five arab countries	BMC Public Health	04-Jun-24	Original / Review	Tunisia	3.5	1	pro-Gaza	0	0	0	0	0
**Zvi Bekerman**[177]	1	Opinon / Politic	Silently Navigating Ethical Paradoxes in the Israel-Hamas Conflict: A Short Note	Bioethical Inquiry	05-Jun-24	Correspondence / Letter / Editorial	Israel	0	NR	pro-Israel	0	0	0	0	0
**Sualeha Shekhani**[178]	2	Opinon / Politic	Amid Explosions in Gaza, The Silence from the Bioethics Community is Deafening	Bioethical Inquiry	05-Jun-24	Original / Review	Pakistan	0	NR	pro-Gaza	1	0	0	1	1
**Ashagrew Liyih**[179]	4	Science	Sentiment analysis of the Hamas? Israel war on YouTube comments using deep learning	Scientific Reports	13-Jun-24	Original / Review	Ethiopia	3.8	1	Neutral	0	0	1	1	0

Author	Author	Topic	Title	Journal	Publication	Publication first author	Country of IF	Journal Q	Journal In	Position In affiliation	Gaza *(referring to the Israeli occupation of Palestine)*	Israel 7	Occupation	October Title / Genocide number Title	Date Citation / Type
**Oqab Jabali**[180]	6	Opinon / Politic	Exploring perceived challenges, adoption, and assessment of Western values of democracy and human rights in Palestine in the 2023 War on Gaza	Scientific Reports	14-Jun-24	Original / Review	Gaza / Palestine	3.8	1	pro-Gaza	1	0	4	0	1
**Saleyha Ahsan**[181]	1	Humanitarian	Israel border closure denying health care to Gazans	The Lancet	15-Jun-24	Correspondence / Letter / Editorial	UK	98.4	1	pro-Gaza	0	0	0	0	0
**Yael Mayer**[182]	7	Science	Mental Health Outcomes of Arab and Jewish Populations in Israel a Month after the Mass Trauma Events of October 7, 2023: A Cross-Sectional Survey of a Representative Sample	Psychiatry Research	15-Jun-24	Original / Review	Israel	4.2	1	pro-Israel	0	1	0	1	0
**Rick Maity**[183]	5	Humanitarian	The ongoing Israel-Hamas conflict: a humanitarian health crisis	Annals Of Medicine & Surgery	20-Jun-24	Correspondence / Letter / Editorial	India	1.7	NR	Neutral	0	0	1	0	0
**Jamil Sawalmeh**[184]	1	Humanitarian	The meagre flow of aid into Gaza is creating a deadly health crisis for Palestinians	BMJ	21-Jun-24	Correspondence / Letter / Editorial	UK	93.6	1	pro-Gaza	1	0	0	0	0
**Ligat Shalev**[185]	7	Psychological	Utilization of psychiatry services in the emergency department following a terror event in Israel	Psychiatry Research	23-Jun-24	Original / Review	Israel	4.2	1	pro-Israel	0	1	0	1	0
**Marion Birch**[186]	1	Opinon / Politic	Who did that? AI-assisted targeting and the lowering of thresholds in Gaza	Medicine, Conflict and Survival	24-Jun-24	Correspondence / Letter / Editorial	UK	0	3	Neutral	0	0	2	0	0
**Houssein H Ayoub**[187]	3	Science	Comparative analysis and evolution of civilian versus combatant mortality ratios in Israel-Gaza conflicts, 2008–2023	Frontiers In Public Health	25-Jun-24	Original / Review	Qatar	3.0	2	pro-Gaza	0	0	1	0	0
**Leslie London**[188]	40	Humanitarian	A call from 40 public health scientists for an end to the continuing humanitarian and environmental catastrophe in Gaza	Environmental Health	28-Jun-24	Correspondence / Letter / Editorial	South Africa	5.3	2	pro-Gaza	1	0	0	1	0
**Nadir Ijaz**[189]	2	Opinon / Politic	Public Health Crisis in Gaza—The Responsibility of US-Based Academic Medical Journals	JAMA	02-Jul-24	Correspondence / Letter / Editorial	USA	63.1	1	pro-Gaza	1	0	0	0	0

Author	Author	Topic	Title	Journal	Publication	Publication first author	Country of IF	Journal Q	Journal In	Position In affiliation	Gaza (*referring*	Israel 7	Occupation October Title	Genocide number Title *to the Israeli occupation of Palestine)*	Date Citation Type
**Rasha Khatib**[190]	3	Humanitarian	Counting the dead in Gaza : difficult but essential	The Lancet	05-Jul-24	Correspondence / Letter / Editorial	USA	98.4	1	Neutral	1	0	0	1	0
**Duha Shellah**[191]	1	Humanitarian	War on Gaza: attacks science are erasing the future	The Lancet	06-Jul-24	Correspondence / Letter / Editorial	Gaza / Palestine	98.4	1	pro-Gaza	1	0	3	0	0
**Shoshana Marmon**[192]	3	Opinon / Politic	A disingenuous one-sided challenge on the Hamas-Israel conflict	The Lancet	11-Jul-24	Correspondence / Letter / Editorial	USA	98.4	1	pro-Israel	0	0	1	0	1
**Zion Hagay**[193]	2	Opinon / Politic	Help Gaza with aid, not false designations	The Lancet	13-Jul-24	Correspondence / Letter / Editorial	Israel	98.4	1	pro-Israel	1	0	0	0	1
**Jerome Teitel**[194]	2	Opinon / Politic	Health-care workers can take the lead in ending the war in Gaza	The Lancet	13-Jul-24	Correspondence / Letter / Editorial	Canada	98.4	1	pro-Israel	1	0	0	0	2
**Eitan Keizman**[195]	8	Science	Cardiac surgery during wartime in Israel	Journal of Cardiothoracic Surgery	15-Jul-24	Original / Review	Israel	1.5	3	pro-Israel	0	1	0	1	0
**Rajiv Tandon**[196]	4	Humanitarian	The Gaza conflict and the role of psychiatry: A call to action	Asian Journal Of Psychiatry	17-Jul-24	Original / Review	USA	3.8	1	pro-Gaza	1	0	0	0	0
**Suha Hamshari**[197]	7	Science	Barriers faced by primary healthcare providers in addressing emergencies in the Northern region of Palestine before and during the Gaza War	BMC Primary Care	17-Jul-24	Original / Review	Gaza / Palestine	2.0	2	pro-Gaza	1	0	2	0	0
**Zairul-Nizam**[198]	2	Opinon / Politic	Deafening Silence of Malaysian Medical and Surgical Fraternities to the Gaza Genocide	Malaysian Orthopaedic Journal	23-Jul-24	Correspondence / Letter / Editorial	Malaysia	0.6	4	pro-Gaza	1	0	0	0	1
**Omar Gammoh**[199]	6	Psychological	The Prevalence and Correlates of Depression, Anxiety, and Insomnia among Camp Residing Palestinian Women Migrants during the Outbreak of the War on Gaza A Cross- Sectional Study from Jordan	Medicina	29-Jul-24	Original / Review	Jordan	2.4	2	pro-Gaza	1	0	8	0	0
**Keren Dopelt**[200]	2	Psychological	War-Related Stress among Israeli College Students Following 7 October 2023 Terror Attack in Israel	Europen Journal Of Investigation In Health, Psychology And Education	30-Jul-24	Original / Review	Israel	0	NR	pro-Israel	0	1	0	1	0

Author	Author	Topic	Title	Journal	Publication	Publication first author	Country of IF	Journal Q	Journal In	Position In affiliation	Gaza *(referring*	Israel 7	Occupation	October Title	Genocide Title	number *to the Israeli occupation of Palestine)*	Date Citation	Type
**Sharon Goldman**[201]	7	Science	October 7th Mass Casualty Attack in Israel	Annals Of Surgery Open	05-Aug-24	Original / Review	Israel	0	NR	pro-Israel	0	1	0	1	0			
**Zohar Mor**[202]	4	Science	Mass killing during terror attack at the Israel-Gaza border and the role of the Ministry of Health in identification of human remains and their release	Forensic Science International	08-Aug-24	Original / Review	Israel	2.2	1	pro-Israel	0	0	0	1	0			
**Rachel Shvartsur**[203]	2	Psychological	Civilians under missile attack: post-traumatic stress disorder among the Jewish and Bedouin population of Southern Israel	Israel Journal Of Health Policy and Research	12-Aug-24	Original / Review	Israel	3.5	1	pro-Israel	0	1	0	1	0			
**Berthold Koletzko**[204]	8	Humanitarian	European Academy of Paediatrics demands protection of children's rights endangered by the ongoing Israel Gaza conflict	Acta Pædiatrica	14-Aug-24	Correspondence / Letter / Editorial	Germany	2.4	1	Neutral	0	0	0	1	0			
**Shimaa Holail**[205]	9	Science	Time-series satellite remote sensing reveals gradually increasing war damage in the Gaza Strip	National Science Review	24-Aug-24	Original / Review	China	16.3	1	pro-Gaza	1	0	2	0	0			
**Rikas Saputra**[206]	4	Humanitarian	Prenatal nutrition and mental health: Long-term effects of hunger in Gaza and health policy implications	Asian Journal Of Psychiatry	30-Aug-24	Correspondence / Letter / Editorial	Indonesia	3.8	1	pro-Gaza	1	0	0	0	0			
**Sharmila Devi**[207] **Khaled Alser**[208]	1	Humanitarian	Calls for Gaza ceasefire to tackle poliovirus	The Lancet	31-Aug-24	Correspondence / Letter / Editorial	Iran	98.4	1	Neutral	1	0	0	0	0			
	9	Science	Trauma care supported through a global telemedicine initiative during the 2023–24 military assault on the Gaza Strip, occupied Palestinian territory: a case series	The Lancet	31-Aug-24	Original / Review	Gaza / Palestine	98.4	1	pro-Gaza	1	0	5	0	0			
**Yassar A Qureshi**[209] **Dalia Zayed**[210]	3	Humanitarian	Excess deaths in Gaza	The Lancet	01-Sep-24	Correspondence / Letter / Editorial	UK	98.4	1	pro-Gaza	1	0	0	0	0			
	3	Humanitarian	Infectious diseases within a wartorn health system: The reemergence of polio in Gaza	New Microbes and New Infections	05-Sep-24	Correspondence / Letter / Editorial	Jordan	2.9	3	pro-Gaza	1	0	0	0	0			

Author	Author Position	Topic	Title	Journal	Publication Date	Publication Type	Country of first author affiliation	Journal IF	Journal Q	Position	Gaza In Title	Israel In Title	Occupation In Title	October 7 / Genocide (referring to the Israeli occupation of Palestine)	number Citation
**Jerome Groopman**[211]	1	Opinon / Politic	Public Health Crisis in Gaza	JAMA	06-Sep-24	Correspondence / Letter / Editorial	USA	63.1	1	pro-Israel	1	0	0	1	0
**David Siscovick**[212]	1	Opinon / Politic	Public Health Crisis in Gaza	JAMA	06-Sep-24	Correspondence / Letter / Editorial	USA	63.1	1	pro-Israel	1	0	0	1	0
**Anand R. Habib**[213]	2	Opinon / Politic	Public Health Crisis in Gaza- reply	JAMA	06-Sep-24	Correspondence / Letter / Editorial	USA	63.1	1	pro-Gaza	1	0	0	0	0
**B. Irfana**[214]	6	Humanitarian	Infection control in conflict zones: practical insights from recent medical missions to Gaza	Journal of Hospital Infection	09-Sep-24	Correspondence / Letter / Editorial	USA	3.9	1	pro-Gaza	1	0	0	0	0
**Talha Burki**[215]	1	Humanitarian	Polio vaccination campaign in Gaza	The Lancet	12-Sep-24	Correspondence / Letter / Editorial	UK	98.4	1	pro-Gaza	1	0	0	1	0
**Eli Jaffe**[216]	3	Science	Prehospital Care Under Fire: Strategies for Evacuating Victims from the Mega Terrorist Attack in Israel on October 7, 2023	Prehospital And Disaster Medicine	18-Sep-24	Correspondence / Letter / Editorial	Israel	2.1	2	pro-Israel	0	1	0	1	0
**Fatima Mohammed**[217]	6	Humanitarian	Defending the right to health in Gaza: a call to action by health workers	Conflict And Health	20-Sep-24	Correspondence / Letter / Editorial	Sudan	3.1	2	pro-Gaza	1	0	0	0	0
**Masako Horino**[218]	8	Science	Food insecurity, dietary inadequacy, and malnutrition in the Gaza Strip: a cross-sectional nutritional assessment of refugee children entering the first grade of UN	Lancet Global Health	23-Sep-24	Original / Review	Jordan	19.9	1	pro-Gaza	1	0	2	1	0
**Lara Nasreddine**[219]	2	Humanitarian	Safeguarding the SDG promise to end hunger and leave no one behind: the plight of children in the Gaza Strip	Lancet Global Health	23-Sep-24	Correspondence / Letter / Editorial	Lebanon	19.9	1	pro-Gaza	1	0	0	0	0
**Smriti Mallapaty**[220]	1	Humanitarian	Gaza: Why is it so hard to establish the death toll?	Nature	24-Sep-24	News	Australia	50.5	1	Neutral	1	0	1	0	0
**Hanna Kienzler**[221]	6	Humanitarian	Unbearable suffering: mental health consequences of the October 2023 Israeli military assault on the Gaza Strip	BMJ Global Health	30-Sep-24	Correspondence / Letter / Editorial	UK	7.1	2	pro-Israel	0	0	1	0	4

References

1. Mahase E. Israel and Gaza: MSF calls for end to “indiscriminate violence and collective punishment”. *BMJ*. 2023;383:2383. doi:10.1136/bmj.p2383.
2. Shahid HJ, Wallace PG. The healthcare community must approach the violence in Israel and Gaza with inclusive compassion. *BMJ*. 2023;383:2645. doi:10.1136/bmj.p2645.
3. Elessi K. Save Gaza residents from imminent catastrophe. *Lancet*. 2023;402(10412):1522-1523. doi:10.1016/S0140-6736(23)02299-7.
4. Mahase E. Gaza: Israel’s hospital evacuation orders are “death sentence for the sick,” says WHO. *BMJ*. 2023;383:2397. doi:10.1136/bmj.p2397.
5. Mahase E. Gaza: “Unprecedented” bombing of hospital leaves hundreds dead and injured. *BMJ*. 2023;383:2423. doi:10.1136/bmj.p2423.
6. Correia T. Two urgent actions related to international health emergencies amid the escalating conflict in Gaza. *Int J Health Plann Manage*. 2024;39(2):160-163. doi:10.1002/hpm.3720.
7. Salmiya MA. Urgent humanitarian call to save lives in Gaza. *Lancet*. 2023;402(10412):1523-1524. doi:10.1016/S0140-6736(23)02333-4.
8. Givaty G, Ovadia YS, Saban M. Insights from the nearest Israeli hospital to the Gaza Strip. *Lancet*. 2023;402(10412):1521-1522. doi:10.1016/S0140-6736(23)02334-6.
9. Abudayya A, Abu Ghali K, Hargreaves S, et al. An urgent call to save and protect lives of vulnerable populations in the Gaza Strip. *Lancet Reg Health Eur*. 2023;35:100767. doi:10.1016/j.lanepe.2023.100767.
10. Seita A, Al-Jadba G. Gaza is facing a humanitarian catastrophe. *Lancet*. 2023;402(10414):1745. doi:10.1016/S0140-6736(23)02401-7.
11. Dyer O. Gaza hospitals are within hours of losing power as fuel embargo continues, say doctors and agencies. *BMJ*. 2023;383:2470. doi:10.1136/bmj.p2470.
12. Buss P, Alcázar S, Souza LE. End the violence and help the victims in Gaza. *Lancet*. 2023;402(10413):1622-1623. doi:10.1016/S01406736(23)02362-0.
13. Zughbur MR. Protect civilians’ lives and health care in Gaza. *Lancet*. 2023;402(10413):1620-1621. doi:10.1016/S0140-6736(23)02402-9.
14. Chabin M. Israel-Hamas war shakes scientific community. *Science*. 2023;382(6669):362. doi:10.1126/science.adl5526.
15. Jabr S, Berger E. Palestine meeting Gaza’s mental health crisis. *Lancet Psychiatry*. 2024;11(1):12. doi:10.1016/S2215-0366(23)00398-X.
16. Horton R. Offline: Israel–Gaza—what comes next? *Lancet*. 2023;402(10412):1511. doi:10.1016/S0140-6736(23)02398-X.
17. Latifi-Pour M, Zavareh SMA. Call for Emergency Aid for Gaza Strip Hospitals and Clinics. *Trauma Monthly*. 2023;28(5):943-944. doi:10.30491/TM.2023.425457.1657.
18. Goodarzi H, Badri T, Javadzadeh HR. War, Forced Displacement, and Lack of Healthcare in the Gaza Strip. *Trauma Monthly*. 2023;28(5):945-946. doi:10.30491/TM.2023.427311.1665.
19. Akhondzadeh S. What is the Tolerance Limit of the Human Society in Face of the Killing of Innocent and Defenseless People of Gaza? Stop the Killing of Children and Infants in Gaza. *Arch Iran Med*. 2023;26(11):606-606. doi:10.34172/aim.2023.89.
20. Sternberg SA, Breuer E. The conflict in Gaza: a view from Israel. *Lancet*. 2023;402(10416):1973-1974. doi:10.1016/S01406736(23)02406-6.
21. Mahase E. Gaza: UN warns that “nowhere is safe” for patients amid “relentless bombing campaign”. *BMJ*. 2023;383:2550. doi:10.1136/bmj.p2550.
22. Rimawi R, Madani N. Gaza’s broken health-care system is compounding the risk of disease. *Nature*. 2023;623(7985):32. doi:10.1038/d41586-023-03349-z.
23. Jones N. “I’m a powder keg”: ousted eLife editor on being fired in wake of Israel-Hamas remarks. *Nature*. 2023;623(7985):13-14. doi:10.1038/d41586-023-03330-w.
24. Akhondzadeh S. Missile Attacks on Al-Ahli Hospital in Gaza and Silence of Reference International Authorities. *J Iran Med Counc*. 2024;7(1):1. doi:10.18502/jimc.v7i1.14195.
25. Horton R. Offline: Gaza’s children—a responsibility to protect. *Lancet*. 2023;402(10413):1609. doi:10.1016/S0140-6736(23)02443-1.
26. Nashwan AJ. A Double Battle: Fighting Cancer in the Shadows of Conflict in Gaza. *Cureus*. 2023;15(11):e48371. doi:10.7759/cureus.48371.

27. Burki T. Last cancer hospital in Gaza. *Lancet Oncol*. 2023;24(12):1313. doi:10.1016/S1470-2045(23)00581-8.
28. Hussein H. Stop violation of international water laws in Gaza. *Nature*. 2023;623(7986):253. doi:10.1038/d41586-023-03461-0.
29. Torfstein A, Cohen A, Birnbaum RY, Kaspi Y. Israel: when reality meets academia. *Nature*. 2023;623(7986):253. doi:10.1038/d41586023-03462-z.
30. Khwaja A, Bell I, Hadley D, Price H, Turnbull C. Gaza: a plea to reclaim our collective humanity. *Lancet*. 2023;402(10416):1975. doi:10.1016/S0140-6736(23)02510-2.
31. Yaqub S, Sparrelid E, Sampaio-Neto J, Lassen K, Wigmore SJ. Israel and Gaza: the killing of civilians must stop. *Lancet*. 2023;402(10417):2069-2070. doi:10.1016/S0140-6736(23)02517-5.
32. Smith J, Abdel-Mannan O, Abuelaish I, Kelly B, Maynard N. Palestine and Israel: for an end to violence and the pursuit of justice. *Lancet*. 2023;402(10416):1974-1975. doi:10.1016/S0140-6736(23)02509-6.
33. Devi S. Health care in Gaza continues to worsen under conflict. *Lancet*. 2023;402(10414):1736. doi:10.1016/S0140-6736(23)02513-8.
34. Taylor L. Stop attacks on Gaza's hospitals, agencies plead, amid collapse of services. *BMJ*. 2023;383:2656. doi:10.1136/bmj.p2656.
35. Levany S, Shahar G, Greenberg D. Calling for an immediate release of captive children in Gaza. *Lancet*. 2023;402(10417):2070-2071. doi:10.1016/S0140-6736(23)02519-9.
36. Howard S. Doctors call on GMC to publish guidance on social media posts on the Israel-Hamas war. *BMJ*. 2023;383:2697. doi:10.1136/bmj.p2697.
37. Aodi F. Israel-Hamas conflict: a call for peace in support of the Gaza population, where women and children are paying the highest price. *Journal of Sex- and Gender-Specific Medicine*. 2023;9(3):105-106.
38. Peleg O, Gendelman L. Internally displaced people amidst war: the Israeli narrative. *The Lancet*. 2023;402(10417):2071-2072. doi:10.1016/S0140-6736(23)02521-7.
39. reporters N news team and freelance. The Israel–Hamas conflict: voices from scientists on the front lines. *Nature*. November 2023. doi:10.1038/d41586-023-03550-0.
40. Shellah D. How much suffering is enough? A look at what is happening in Gaza. *Lancet*. 2023;402(10417):2072. doi:10.1016/S01406736(23)02555-2.
41. Bjertness E, Bjertness MS, Nyquist CB, Kromberg M, Elessi K, Lien L. Save the remaining people of Gaza—save the children. *Lancet*. 2023;402(10417):2072-2073. doi:10.1016/S0140-6736(23)02556-4.
42. Uvais NA, Ashfaq U Rahman AM. Jaspersian Principles for Achieving Lasting Peace in the Palestine-Israel Conflict. *Asian J Psychiatr*. 2024;91:103850. doi:10.1016/j.ajp.2023.103850.
43. Kadivar M. In Search of a Safe Place in Gaza for Children Stop Violence Against Children. *J Iran Med Counc*. 2024;7(1):2-4. doi:10.18502/jimc.v7i1.14196.
44. Rees SJ, Moussa B. Invisible wounds of the Israel–Gaza war in Australia. *Med J Aust*. 2024;220(1):4-6. doi:10.5694/mja2.52168.
45. Dyer O. More Gaza hospitals come under fire as Israeli forces search al-Shifa. *BMJ*. 2023;383:2756. doi:10.1136/bmj.p2756.
46. Beiraghdar F, Momeni J, Hosseini E, Panahi Y, Negah SS. Health Crisis in Gaza: The Urgent Need for International Action. *Iran J Public Health*. 2023;52(12):2478-2483. doi:10.18502/ijph.v52i12.14309.
47. Nacasch N, Shoenfeld N, Wul I, Polliack M, Weiser M. Prevention of Post-traumatic stress disorder by Early Psychological Interventions in Israel Following the October 7th Massacre. *Isr Med Assoc J*. 2023;25(11):724-728.
48. Moussally K, Abu-Sittah G, Gomez FG, Fayad AA, Farra A. Antimicrobial resistance in the ongoing Gaza war: a silent threat. *Lancet*. 2023;402(10416):1972-1973. doi:10.1016/S0140-6736(23)02508-4.
49. Jamaluddine Z, Checchi F, Campbell OMR. Excess mortality in Gaza: Oct 7–26, 2023. *Lancet*. 2023;402(10418):2189-2190. doi:10.1016/S0140-6736(23)02640-5.
50. Salmiya MA. Gaza hospitals: military siege and bombings. *Lancet*. 2023;402(10418):2191. doi:10.1016/S0140-6736(23)02566-7.

51. Elyoseph Z, Hadar-Shoval D, Angert T, et al. Mental health volunteers after the Oct 7 Gaza border crisis in Israel: silent warriors. *Lancet Psychiatry*. 2024;11(1):10-12. doi:10.1016/S2215-0366(23)00369-3.
52. Qurbani K, Ahmed SK, Hussein S, Omar RM. Urgent attention needed for the mental health of people in Gaza: A call for global action. *Asian J Psychiatr*. 2024;91:103851. doi:10.1016/j.ajp.2023.103851.
53. Launer J. John Launer: Israel and Gaza—recognising shared human values. *BMJ*. 2023;383:2768. doi:10.1136/bmj.p2768.
54. Musa A, Crawley J, Haj-Hassan T, Inglis R, Maynard N. Gaza, 9 years on: a humanitarian catastrophe. *Lancet*. 2023;402(10419):22922293. doi:10.1016/S0140-6736(23)02639-9.
55. Nerlander MP, Klein A, Herbst R, Jaffe E. Strengthening Preparedness Against Terror: A Bystander Training Concept by Israel's Emergency Medical Services. *Am Surg*. 2024;90(4):489-493. doi:10.1177/00031348231219257.
56. Saad HB, Ghorbel IB, Saad SB, et al. Gaza Genocide: Breaking the silence. *Tunis Med*. 2023;101(11):787-788.
57. Al-Mandhari A, Peeperkorn R, Al-Shorbaji F, Akbar B, Kamil AM, Brennan R. Gaza disaster: we need a permanent ceasefire, now! *East Mediterr Health J*. 2023;29(12):919-920. doi:10.26719/2023.29.12.919.
58. Salai M, Sandhaus Y, Golik A, et al. Desecration by Hamas of the Holy Ten Commandments Embedded in Medical Education during the Iron Swords War in Gaza. *Isr Med Assoc J*. 2023;25(12):791-792.
59. Levin G, Meyer R, Brezinov Y. Analysis of Scientific Publications on the Gaza-Israeli Conflict. *Isr Med Assoc J*. 2023;25(12):795-796.
60. Kugel C, Arnheim D, Dotan A, Furman M, Shoenfeld Y. Forensic Frontiers: Navigating Complex Challenges of a Large-scale Invasion by Armed Hamas Terrorists in Southern Israel. *Isr Med Assoc J*. 2023;25(12):787-790.
61. Mahmoud H, Abuzerr S. State of the health-care system in Gaza during the Israel–Hamas war. *Lancet*. 2023;402(10419):2294. doi:10.1016/S0140-6736(23)02634-X.
62. Soni S. Gaza and international law: The global obligation to protect life and health. *S Afr J Bioeth Law*. 2023;16(3):80-81. doi:10.7196/SAJBL.2023.v16i3.1764.
63. Mahomed S. When sanctuaries of humanity turn into corridors of horror: The destruction of healthcare in Gaza. *S Afr J Bioeth Law*. 2023;16(3). doi:10.7196/SAJBL.2023.v16i3.1732.
64. Arana-Cedeño M. Hospitals, health and death in Gaza. *Soc Med*. 2023;16(3):155-159.
65. Mahase E. Conditions in Gaza are ripe for "epidemics and public health disaster," UN warns. *BMJ*. 2023;383:2887. doi:10.1136/bmj.p2887.
66. Ahmed SK. Addressing the Effects of War on Gaza's Healthcare System. *Cureus*. 2023;15(12):e50036. doi:10.7759/cureus.50036.
67. Huynh BQ, Chin ET, Spiegel PB. No evidence of inflated mortality reporting from the Gaza Ministry of Health. *Lancet*. 2024;403(10421):23-24. doi:10.1016/S0140-6736(23)02713-7.
68. Hussein-Sabbah S. Pregnant women in Gaza face perilous conditions as maternity services and infrastructure crumble. *BMJ*. 2023;383:2895. doi:10.1136/bmj.p2895.
69. Banerjee A. The conflict in Gaza—are data ever enough? *Lancet*. 2024;403(10421):27. doi:10.1016/S0140-6736(23)02637-5.
70. Salam. There is no way to leave Gaza. *Lancet*. 2024;403(10421):26-27. doi:10.1016/S0140-6736(23)02676-4.
71. Horton R. Offline: "My brothers and sisters in Israel and Palestine." *Lancet*. 2023;402(10418):2180. doi:10.1016/S0140-6736(23)02745-9.
72. WHO's Executive Board adopts resolution on access for life-saving aid into Gaza and respect for laws of war. https://www.who.int/news/item/10-12-2023-who-s-executive-board-adopts-resolution-on-access-for-life-saving-aid-into-gaza-and-respect-forlaws-of-war. Accessed July 28, 2024.
73. Zarocostas J. WHO Executive Board calls for action in Gaza. *Lancet*. 2023;402(10419):2278-2279. doi:10.1016/S0140-6736(23)02794-0.
74. Shepherd A. Help women in Gaza and across the globe. *BMJ*. 2023;383:2959. doi:10.1136/bmj.p2959.

75. Clarfield AM. Older persons, new challenges: Caring for vulnerable persons during wartime in Israel. *J Am Geriatr Soc*. December 2023. doi:10.1111/jgs.18710.
76. Teitel J. Israel and Gaza: a quick end to a tragic but necessary war. *Lancet*. 2024;403(10422):143. doi:10.1016/S0140-6736(23)02567-9.
77. Dhai A. The situation in Gaza – will cruelty and hatred triumph? *S Afr J Bioeth Law*. 2023;16(3):74. doi:10.7196/SAJBL.2023.v16i3.1819.
78. Feder G, Khan A, Jewell D, Jameel S. Responding to the war in Israel and Palestine. *Br J Gen Pract*. 2024;74(739):76. doi:10.3399/bjgp24X736257.
79. Alokaily F. War and Health Crisis in Gaza. *Saudi Med J*. 2024;45(1):9. doi:10.15537/smj.2023.44.1.20240012.
80. Muthumani A. Safeguarding children through pediatric surgical care in war and humanitarian settings: a call to action for pediatric patients in Gaza. *World J Pediatr Surg*. 2024;7(1):e000719. doi:10.1136/wjps-2023-000719.
81. Codish S, Frenkel A, Klein M, Geftler A, Dreiher J, Schwarzfuchs D. October 7th 2023 attacks in Israel: frontline experience of a single tertiary center. *Intensive Care Med*. 2024;50(2):308-310. doi:10.1007/s00134-023-07293-4.
82. Levi-Belz Y, Groweiss Y, Blank C, Neria Y. PTSD, depression, and anxiety after the October 7, 2023 attack in Israel: a nationwide prospective study. *EClinicalMedicine*. 2024;68:102418. doi:10.1016/j.eclinm.2023.102418.
83. Mahase E. Gaza: WHO describes “sickening scenes” in hospital as Israeli military intensifies activity. *BMJ*. 2024;384:q43. doi:10.1136/bmj.q43.
84. Elnakib S, Fair M, Mayrhofer E, Afifi M, Jamaluddine Z. Pregnant women in Gaza require urgent protection. *Lancet*. 2024;403(10423):244. doi:10.1016/S0140-6736(23)02835-0.
85. Zivot JB, Katz NT, Deckelbaum RJ, Lantos JD, Teitel JM. Gaza and the complexity and context of suffering. *Lancet*. 2024;403(10423):244-245. doi:10.1016/S0140-6736(23)02836-2.
86. Attia MAH. A call for the initiation of the forensic humanitarian action in Gaza. *Egyptian Journal of Forensic Sciences*. 2024;14(1). doi:10.1186/s41935-023-00384-9.
87. Bagheri Lankarani K. The War of Gaza and International Laws. *Shiraz E Med J*. 2024;25(1). doi:10.5812/semj-144177.
88. Taylor L. Allow more aid into Gaza or risk famine, warns WHO. *BMJ*. 2024;384:q115. doi:10.1136/bmj.q115.
89. Gostin LO, Goodwin MB. Wars in Gaza and Beyond: Why Protecting the Sacredness of Health Matters. *JAMA*. 2024;331(3):191-192. doi:10.1001/jama.2023.26391.
90. Coghlan R, Smith J, Alser O. Morphine for Gaza? Limits of care during genocidal violence. *Lancet*. 2024;403(10424):350-351. doi:10.1016/S0140-6736(24)00014-X.
91. Souza LE, Buss P, Alcázar S. Wars are never necessary: Gaza is the best example. *Lancet*. 2024;403(10424):349-350. doi:10.1016/S0140-6736(24)00013-8.
92. Arawi T. War on healthcare services in Gaza. *Indian J Med Ethics*. 2024;IX(2):130-135. doi:10.20529/IJME.2024.004.
93. Nsutebu E, AlDhaheri F, Albasata H, Aldhanhani H, Hussein S, Al Hammadi A. Gaza-ceasefire and prepare for a surge in infectious diseases! *Postgrad Med J*. February 2024:qgae022. doi:10.1093/postmj/qgae022.
94. Shellah D. War on Gaza: the impossible duty to care for the critically ill. *Intensive Care Med*. 2024;50(2):311-313. doi:10.1007/s00134023-07309-z.
95. Mahase E. Gaza-Israel conflict: Hundreds of medics are killed or arrested after intense attacks on healthcare facilities. *BMJ*. 2024;384:q203. doi:10.1136/bmj.q203.
96. Looi M-K. The BMJ Appeal 2023-24: Gaza’s mothers and babies need your help. *BMJ*. 2024;384:q172. doi:10.1136/bmj.q172.
97. Alpert EA, Assaf J, Nama A, Pliner R, Jaffe E. Secondary Ambulance Transfers During the Mass-Casualty Terrorist Attack in Israel on October 7, 2023. *Prehosp Disaster Med*. 2024;39(2):224-227. doi:10.1017/S1049023X24000153.
98. Almog O, Benov A, Beer Z, Sirotkin T, Shental O, Glassberg E. Deploying whole blood to the battlefield—The Israel Defense Forces Medical Corps initial experience during the 2023 war. *Transfusion*. 2024. doi:10.1111/trf.17718.

99. Taha AM, Mahmoud H, Nada SA, Abuzerr S. Controlling the alarming rise in infectious diseases among children younger than 5 years in Gaza during the war. *Lancet Infect Dis*. 2024;24(4):e211. doi:10.1016/S1473-3099(24)00067-7.
100. Greenland P, Lakser O, Lipschutz L. Importance of a broader view of the Hamas–Israel war. *BMJ Glob Health*. 2024;9(2). doi:10.1136/bmjgh-2023-014378.
101. Lazar A, Gewirtz-Meydan A, Rosenbaum TY. War-Time Stress and Sexual Well-Being in Israel. *Int J Sex Health*. 2024;36(1):1-14. doi:10.1080/19317611.2024.2317169.
102. Ayalon L, Okun S, Cohn-Schwartz E, Sagi D. On Intergenerational Conflict and Solidarity at Times of Terror and War in Israel: The Case of Late-Life Physical Vulnerabilities and Emotional Resilience. *Am J Geriatr Psychiatry*. 2024;32(5):639-641. doi:10.1016/j.jagp.2024.01.222.
103. Ben Saad H. Urgent humanitarian appeal: Protecting the lives of women and children in the Gaza Strip (Palestine). *J Public Health Res*. 2024;13(1):22799036241229312. doi:10.1177/22799036241229312.
104. Kearney JE, Thiel N, El-Taher A, et al. Conflicts in Gaza and around the world create a perfect storm for infectious disease outbreaks. *PLOS Glob Public Health*. 2024;4(2):e0002927. doi:10.1371/journal.pgph.0002927.
105. Taha AM, Sabet C, Nada SA, Abuzerr S, Nguyen D. Addressing the mental health crisis among children in Gaza. *Lancet Psychiatry*. 2024;11(4):249-250. doi:10.1016/S2215-0366(24)00036-1.
106. Poole DN, Andersen D, Raymond NA, et al. Damage to medical complexes in the Gaza Strip during the Israel–Hamas war: a geospatial analysis. *BMJ Glob Health*. 2024;9(4). doi:10.1136/bmjgh-2023-014768.
107. Qandil M. Gaza: providing emergency care under fire. *Emerg Med J*. 2024;41(4):272-273. doi:10.1136/emermed-2024-213963.
108. Boukari Y, Kadir A, Waterston T, et al. Gaza, armed conflict and child health. *BMJ Paediatr Open*. 2024;8(1). doi:10.1136/bmjpo-2023002407.
109. Al-Jadba G, Zeidan W, Spiegel PB, Shaer T, Najjar S, Seita A. UNRWA at the frontlines: managing health care in Gaza during catastrophe. *Lancet*. 2024;403(10428):723-726. doi:10.1016/S0140-6736(24)00230-7.
110. Khan MS, Tinua AT. Israel–Palestine: dehumanisation and silencing. *Lancet*. 2024;403(10429):805-806. doi:10.1016/S01406736(24)00043-6.
111. Mahase E. Gaza: Doctors of the World office destroyed, as medics are forced to amputate without anaesthetic. *BMJ*. 2024;384:q429. doi:10.1136/bmj.q429.
112. Looi M-K. Gaza: "No health system left," says MSF. *BMJ*. 2024;384:q484. doi:10.1136/bmj.q484.
113. Razum O, Barach P, Bochenek T, et al. Statement of the ASPHER Task Force on War and Public Health on the Conflict in Israel/Palestine. *Public Health Rev*. 2024;45:1607047. doi:10.3389/phrs.2024.1607047.
114. Lederman Z, Davidovitch N, Lederman S. Making a Case for Appropriate and Humane Treatment of Hamas Belligerents in Israel. *Am J Bioeth*. 2024;24(2):8-10. doi:10.1080/15265161.2023.2296411.
115. Balkhy H. As sickness, hunger, and disease spread, Gaza's health system faces increasing threats. *BMJ*. 2024. doi:10.1136/bmj.q519.
116. Jaffe E, Wacht O, Davidovitch N, et al. Managing a Mega Mass Casualty Event by a Civilian Emergency Medical Services Agency: Lessons From the First Day of the 2023 Hamas-Israel War. *Int J Public Health*. 2024;69:1606907. doi:10.3389/ijph.2024.1606907.
117. Mendlovic J, Dadon Y, Mimouni FB. A Statistical Approach to the High Mortality Rate of Israeli Citizens Held Hostage in Gaza. *Isr Med Assoc J*. 2024;26(3):141-142.
118. Albhaisi S. The impossible mission: to save and support science in Gaza. *Lancet*. 2024;403(10429):806. doi:10.1016/S01406736(24)00238-1.
119. Kaim A, Tov MS, Kimhi S, Marciano H, Eshel Y, Adini B. A longitudinal study of societal resilience and its predictors during the IsraelGaza war. *Appl Psychol Health Well Being*. March 2024. doi:10.1111/aphw.12539.
120. Abed Alah M. Echoes of conflict: the enduring mental health struggle of Gaza's healthcare workers. *Confl Health*. 2024;18(1):21. doi:10.1186/s13031-024-00577-6.

121. Bird SM. Threat level in the Gaza Strip: fatalities per 1000 person-years. *Lancet*. 2024;403(10432):1139-1140. doi:10.1016/S01406736(24)00402-1.
122. Abbas S, Mitchell L. Australian medical leadership's silence on Gaza is a moral failure. *Lancet*. 2024;403(10432):1138-1139. doi:10.1016/S0140-6736(24)00401-X.
123. Khoodoruth MAS, Khoodoruth WNC-K. From past famines to present crises: The epigenetic impact of maternal malnutrition on offspring health in Gaza. *Asian J Psychiatr*. 2024;95:103999. doi:10.1016/j.ajp.2024.103999.
124. Asi Y, Mills D, Greenough PG, et al. "Nowhere and no one is safe": spatial analysis of damage to critical civilian infrastructure in the Gaza Strip during the first phase of the Israeli military campaign, 7 October to 22 November 2023. *Confl Health*. 2024;18(1):24. doi:10.1186/s13031-024-00580-x.
125. Mahase E. Gaza: Children dying of starvation as UN food trucks turned away, say aid organisations. *BMJ*. 2024;384:q619. doi:10.1136/bmj.q619.
126. Sabet C, Nguyen D, Nada SA, Abuzerr S, Taha AM. Women and girls in Gaza face increasingly dire physical and mental health challenges. *BMJ*. 2024;384:q625. doi:10.1136/bmj.q625.
127. Gostin LO, Goodwin MB. International Humanitarian Law in the Israeli-Gaza Conflict-Reply. *JAMA*. 2024;331(15):1330. doi:10.1001/jama.2024.4426.
128. Mahase E. Gaza: BMA calls for urgent investigation into mistreatment of doctors. *BMJ*. 2024;384:q658. doi:10.1136/bmj.q658.
129. Hasson-Ohayon I, Horesh D. A unique combination of horror and longing: Traumatic grief in post–October 7, 2023, Israel. *J Trauma Stress*. 2024;37(2):348-351. doi:10.1002/jts.23026.
130. Yaqub S, Edwin B, Hammoud Z, et al. Gaza War: Too many citizens being killed. *Br J Surg*. 2024;111(4). doi:10.1093/bjs/znae094.
131. Salem Gammoh O, Alqudah A, Alotaibi B. Factors associated with insomnia and fatigue symptoms during the outbreak of Oct.7th war on Gaza: A study from Jordan. *Prev Med Rep*. 2024;41:102685. doi:10.1016/j.pmedr.2024.102685.
132. Shrira A, Palgi Y. Age differences in acute stress and PTSD symptoms during the 2023 Israel-Hamas war: Preliminary findings. *J Psychiatr Res*. 2024;173:111-114. doi:10.1016/j.jpsychires.2024.03.003.
133. Levi H, Givaty G, Ovadia YS, Alon Y, Saban M. Evaluating emergency response at a hospital near the Gaza border within 24 h of increased conflict. *BMC Emerg Med*. 2024;24(1):47. doi:10.1186/s12873-024-00964-5.
134. Mahase E. Gaza: "Hospitals should never be battlegrounds," says WHO amid raids on al-Shifa hospital. *BMJ*. 2024;384:q718. doi:10.1136/bmj.q718.
135. Komesaroff PA. It is Not Too Late for Reconciliation Between Israel and Palestine, Even in the Darkest Hour. *J Bioeth Inq*. 2024;21(1):29-45. doi:10.1007/s11673-024-10347-x.
136. Menon K. Responding to the war in Israel and Palestine. *Br J Gen Pract*. 2024;74(741):154. doi:10.3399/bjgp24X736785.
137. Ayalon L, Cohn-Schwartz E, Sagi D. Global Conflict and the Plight of Older Persons: Lessons From Israel. *Am J Geriatr Psychiatry*. 2024;32(4):509-511. doi:10.1016/j.jagp.2023.11.012.
138. Strous RD, Monovich Y. The ethics of psychiatric management in times of disaster and war: experiences from Israel after the Oct 7 attack. *Lancet Psychiatry*. 2024;11(4):242-244. doi:10.1016/S2215-0366(24)00057-9.
139. Hassan F, London L, Manjra S. The global health community must call for an immediate ceasefire and unrestricted humanitarian aid in Gaza. *BMJ*. 2024;385:q782. doi:10.1136/bmj.q782.
140. Lohana AC, Gulati A, Kumar J, Shivani F, Kumar D. The Silent Victims: How the Israel-Palestine War Impacts the Management of Chronic Kidney Disease and End-Stage Renal Disease Patients. *Cureus*. 2024;16(3):e55488. doi:10.7759/cureus.55488.
141. Helpman L, Saragosti GY, Oberman M, Avrahami I, Horesh D. Creating new life while lives are lost: birth in the face of war in Israel after the October 7 attacks. *J Reprod Infant Psychol*. 2024;42(3):377-380. doi:10.1080/02646838.2024.2335782.
142. Mahase E. Gaza: Muslim doctors in UK feel censored and targeted for expressing concern over humanitarian crisis. *BMJ*. 2024;385:q805. doi:10.1136/bmj.q805.

143. Engebretsen E, Baker M. The Rhetoric of Decolonizing Global Health Fails to Address the Reality of Settler Colonialism: Gaza as a Case in Point. *Int J Health Policy Manag*. 2024;13:8419. doi:10.34172/ijhpm.2024.8419.
144. Roth S, Wald HS, Spence NZ, Oratz R, Schwartz DM. Hypocrisy of moral imperatives in the Israel–Hamas war. *Lancet*. 2024;403(10436):1542. doi:10.1016/S0140-6736(24)00241-1.
145. Moscatelli A, Spiga G, Rosati U, Fanton C, Ligarotti GK. Medical evacuation challenges of children from Gaza. *Lancet*. 2024;403(10436):1540. doi:10.1016/S0140-6736(24)00471-9.
146. Landesman LY, Rubinstein RA, Englander BS. Peacebuilding Through Cooperation in Health Care and Public Health Between Israel and Palestine. *J Public Health Manag Pract*. 2024;30(3):315-318. doi:10.1097/PHH.0000000000001919.
147. Levi-Belz Y, Shoval-Zuckerman Y, Blank C, Groweiss Y, Neria Y. The moderating role of belongingness in the contribution of depression to suicide ideation following the October 7, 2023, terrorist attack in Israel: A nationwide prospective study. *J Affect Disord*. 2024;356:292-299. doi:10.1016/j.jad.2024.04.055.
148. Feingold D, Neria Y, Bitan DT. PTSD, distress and substance use in the aftermath of October 7th, 2023, terror attacks in Southern Israel. *J Psychiatr Res*. 2024;174:153-158. doi:10.1016/j.jpsychires.2024.04.022.
149. Brazg Ferro L, Corn BW, Goldzweig G, Sultan M, Shekel E, Sapir E. Radiation Therapy Delivery During the 2023 Israel-Hamas War: Trust Prevails Over Fear. *Adv Radiat Oncol*. 2024;9(7):101514. doi:10.1016/j.adro.2024.101514.
150. Sheinfeld Gorin SN. International Humanitarian Law in the Israeli-Gaza Conflict. *JAMA*. 2024;331(15):1328-1329. doi:10.1001/jama.2024.4435.
151. Adler NR, Chait-Rubinek L, Grant-Kels JM. International Humanitarian Law in the Israeli-Gaza Conflict. *JAMA*. 2024;331(15):13271328. doi:10.1001/jama.2024.4438.
152. Richter ED, Berry EM, Rivkind AI. International Humanitarian Law in the Israeli-Gaza Conflict. *JAMA*. 2024;331(15):1329-1330. doi:10.1001/jama.2024.4429.
153. Kels CG. International Humanitarian Law in the Israeli-Gaza Conflict. *JAMA*. 2024;331(15):1329. doi:10.1001/jama.2024.4432.
154. Gammoh O, Ennab W. The prevalence and correlates of PTSD, insomnia, and fatigue among people with epilepsy during Oct.7th war on Gaza: A study from Jordan. *Epilepsy Behav*. 2024;155:109768. doi:10.1016/j.yebeh.2024.109768.
155. Abuzerr S, Al-Jawaldeh A, Ashour Y, Zinszer K, El Bilbeisi AH. The silent crisis: effect of malnutrition and dehydration on children in Gaza during the war. *Front Nutr*. 2024;11:1395903. doi:10.3389/fnut.2024.1395903.
156. Al-Jamal A. Gaza. *Lancet Psychiatry*. 2024;11(6):414. doi:10.1016/S2215-0366(24)00128-7.
157. Gautier Hirsch A. Peace between Israel and Palestine? *Torture*. 2024;34(1):148-149. doi:10.7146/torture.v34i1.144143.
158. de Waal A. Famine in Gaza: an example of the global humanitarian crisis. *Am J Clin Nutr*. 2024. doi:10.1016/j.ajcnut.2024.04.015.
159. Paltiel O, Manor O, Calderon Margalit R, et al. Children on the Gaza-Israel Border: Victims of War. *Public Health Rev*. 2024;45:1607192. doi:10.3389/phrs.2024.1607192.
160. Houminer Klepar N, Davidovitch N, Dopelt K. Emotional Eating among College Students in Israel: A Study during Times of War. *Foods*. 2024;13(9). doi:10.3390/foods13091347.
161. Maytles R, Shrira A. Caring for Internally Displaced Older Adult Israelis during the 2023 Israel-Hamas War. *Am J Geriatr Psychiatry*. 2024;32(5):642-643. doi:10.1016/j.jagp.2024.01.224.
162. Razum O, Agha H, Davidovitch N, McCall T, Shapira S. Gaza Ceasefire: Improve WASH, Promote Cooperation. *Int J Public Health*. 2024;69:1607412. doi:10.3389/ijph.2024.1607412.

163. Ashour Y, Jlambo A, Abuzerr S. Patients in Gaza with chronic conditions need urgent interventions. *Lancet*. 2024;403(10439):18471848. doi:10.1016/S0140-6736(24)00705-0.
164. Abuzerr S, Zinser K. Amplifying the voices of women in the Gaza Strip. *Lancet*. 2024;403(10439):1848-1849. doi:10.1016/S01406736(24)00680-9.
165. Pitcho S. The stuff that nightmares are made of: Israeli dreams in times of the Israel-Hamas war. *Death Stud*. 2024. doi:10.1080/07481187.2024.2348054.
166. Sah S, Dawas K. Israel is using starvation as a weapon of war in Gaza. *BMJ*. 2024;385:q1018. doi:10.1136/bmj.q1018.
167. Shahvisi A. The ethical is political: Israel's production of health scarcity in Gaza. *J Med Ethics*. 2024;50(5):289-291. doi:10.1136/jme2024-110064.
168. Wispelwey B, Mills D, Asi YM, Hammoudeh W, Kunichoff D, Ahmed AK. Civilian mortality and damage to medical facilities in Gaza. *BMJ Glob Health*. 2024;9(5). doi:10.1136/bmjgh-2023-014756.
169. Lloyd G. Seeing Gaza: Objectivity and Emotion. *J Bioeth Inq*. May 2024. doi:10.1007/s11673-024-10362-y.
170. Al-Maghaireh D, Shawish NS, Alsaqer K, et al. Acute Stress Disorders Among Jordanian Adolescents After Watching Gaza News Footage on Social Media. *J Multidiscip Healthc*. 2024;17:2521-2533. doi:10.2147/JMDH.S461333.
171. Segev R, Videl H, Spitz A. Nurses under fire: Insights from testimonies of community nurses and midwives in nonhospital settings in the southern Israel conflict zone. *Res Nurs Health*. 2024;47(5):513-521. doi:10.1002/nur.22402.
172. Yerramilli P. Refeeding in crisis settings: Implications on health care needs in Gaza. *PLOS Glob Public Health*. 2024;4(5):e0003280. doi:10.1371/journal.pgph.0003280.
173. Salmiya MA. Stop the Gaza genocide immediately. *Lancet*. 2024;403(10441):2286-2287. doi:10.1016/S0140-6736(24)00135-1.
174. Levkovich I, Labes M. "I Felt a Sense of Mission during Moments of Crisis": Mental Health Professionals' Perspectives on Their Initial Treatment of Evacuees during the Israel–Hamas Conflict. *Healthcare*. 2024;12(11):1098. doi:10.3390/healthcare12111098.
175. Blanchet K, Najem M, Shadid L, et al. Rebuilding the health sector in Gaza: alternative humanitarian voices. *Confl Health*. 2024;18(1):42. doi:10.1186/s13031-024-00599-0.
176. Fekih-Romdhane F, Helmy M, Alhuwailah A, et al. Mediating effect of depression and acute stress between exposure to Israel-Gaza war media coverage and insomnia: a multinational study from five arab countries. *BMC Public Health*. 2024;24(1):1498. doi:10.1186/s12889-02418996-8.
177. Bekerman Z. Silently Navigating Ethical Paradoxes in the Israel-Hamas Conflict: A Short Note. *J Bioeth Inq*. June 2024. doi:10.1007/s11673-024-10348-w.
178. Shekhani S, Jafarey A. Amid Explosions in Gaza, The Silence from the Bioethics Community is Deafening. *J Bioeth Inq*. June 2024. doi:10.1007/s11673-024-10364-w.
179. Liyih A, Anagaw S, Yibeyin M, Tehone Y. Sentiment analysis of the Hamas-Israel war on YouTube comments using deep learning. *Sci Rep*. 2024;14(1):13647. doi:10.1038/s41598-024-63367-3.
180. Jabali O, Sleem H, Ayyoub AA, Saeedi M, Alawneh Y, Ishtaiyeh M. Exploring perceived challenges, adoption, and assessment of Western values of democracy and human rights in Palestine in the 2023 War on Gaza. *Sci Rep*. 2024;14(1):13735. doi:10.1038/s41598-02460147-x.
181. Ahsan S. Israel border closure denying health care to Gazans. *Lancet*. 2024;403(10444):2581. doi:10.1016/S0140-6736(24)01240-6.
182. Mayer Y, Shiffman N, Bergmann E, et al. Mental Health Outcomes of Arab and Jewish Populations in Israel a Month after the Mass Trauma Events of October 7, 2023: A Cross-Sectional Survey of a Representative Sample. *Psychiatry Res*. 2024;339:116042. doi:10.1016/j.psychres.2024.116042.
183. Maity R, Kumar H, Dhali A, Biswas J, Kumar B. The ongoing Israel-Hamas conflict: a humanitarian health crisis. *Ann Med Surg (Lond)*. 2024;86(8):4313-4315. doi:10.1097/MS9.0000000000002242.
184. Sawalmeh J. The meagre flow of aid into Gaza is creating a deadly health crisis for Palestinians. *BMJ*. 2024;385:q1390. doi:10.1136/bmj.q1390.

185. Shalev L, Avni A, Tene O, et al. Utilization of psychiatry services in the emergency department following a terror event in Israel. *Psychiatry Res*. 2024;339:116059. doi:10.1016/j.psychres.2024.116059.
186. Birch M. Who did that? AI assisted targeting and the lowering of thresholds in Gaza. *Med Confl Surviv*. 2024;40(2):97-100. doi:10.1080/13623699.2024.2364937.
187. Ayoub HH, Chemaitelly H, Abu-Raddad LJ. Comparative analysis and evolution of civilian versus combatant mortality ratios in IsraelGaza conflicts, 2008-2023. *Front Public Health*. 2024;12:1359189. doi:10.3389/fpubh.2024.1359189.
188. London L, Watterson A, Mergler D, et al. A call from 40 public health scientists for an end to the continuing humanitarian and environmental catastrophe in Gaza. *Environ Health*. 2024;23(1):59. doi:10.1186/s12940-024-01097-9.
189. Ijaz N, Habib AR. Public Health Crisis in Gaza-The Responsibility of US-Based Academic Medical Journals. *JAMA*. 2024;332(1):13-14. doi:10.1001/jama.2024.10766.
190. Khatib R, McKee M, Yusuf S. Counting the dead in Gaza: difficult but essential. *Lancet*. 2024;404(10449):237-238. doi:10.1016/S01406736(24)01169-3.
191. Shellah D. War on Gaza: attacks on science are erasing the future. *Lancet*. 2024;404(10447):27-28. doi:10.1016/S0140-6736(24)00767-0.
192. Marmon S, Schwartz DM, Wald HS. A disingenuous one-sided challenge on the Hamas-Israel conflict. *Lancet*. 2024;404(10448):124. doi:10.1016/S0140-6736(24)01253-4.
193. Hagay Z, Borow M. Help Gaza with aid, not false designations. *Lancet*. 2024;404(10448):122. doi:10.1016/S0140-6736(24)01250-9.
194. Teitel J, Zivot J. Health-care workers can take the lead in ending the war in Gaza. *Lancet*. 2024;404(10448):122-123. doi:10.1016/S01406736(24)01251-0.
195. Keizman E, Jamal T, Sarantsev I, et al. Cardiac surgery during wartime in Israel. *J Cardiothorac Surg*. 2024;19(1):446. doi:10.1186/s13019-024-02907-4.
196. Tandon R, Keshavan MS, Javed A, Rao GP. The Gaza conflict and the role of psychiatry: A call to action. *Asian J Psychiatr*. 2024;98:104160. doi:10.1016/j.ajp.2024.104160.
197. Hamshari S, Hamadneh S, Ghneem M, et al. Barriers faced by primary healthcare providers in addressing emergencies in the Northern region of Palestine before and during the Gaza war. *BMC Prim Care*. 2024;25(1):261. doi:10.1186/s12875-024-02512-3.
198. Zairul-Nizam ZF, Ibrahim NA. Deafening Silence of Malaysian Medical and Surgical Fraternities to the Gaza Genocide. *Malays Orthop J*. 2024;18(2):95-96. doi:10.5704/MOJ.2407.015.
199. Gammoh O, Sayaheen B, Alsous M, Al-Smadi A, Al-Jaidi B, Aljabali AAA. The Prevalence and Correlates of Depression, Anxiety, and Insomnia among Camp Residing Palestinian Women Migrants during the Outbreak of the War on Gaza: A Cross-Sectional Study from Jordan. *Medicina (Kaunas)*. 2024;60(8). doi:10.3390/medicina60081228.
200. Dopelt K, Houminer-Klepar N. War-Related Stress among Israeli College Students Following 7 October 2023 Terror Attack in Israel. *Eur J Investig Health Psychol Educ*. 2024;14(8):2175-2186. doi:10.3390/ejihpe14080145.
201. Goldman S, Lipsky AM, Radimislensky I, et al. October 7th Mass Casualty Attack in Israel: Injury Profiles of Hospitalized Casualties. *Ann Surg Open*. 2024;5(3):e481. doi:10.1097/AS9.0000000000000481.
202. Mor Z, Kugel C, Mizrahi H, Kaliner E. Mass killing during terror attack at the Israel-Gaza border and the role of the Ministry of Health in identification of human remains and their release. *Forensic Sci Int*. 2024;363:112188. doi:10.1016/j.forsciint.2024.112188.
203. Shvartsur R, Savitsky B. Civilians under missile attack: post-traumatic stress disorder among the Jewish and Bedouin population of Southern Israel. *Isr J Health Policy Res*. 2024;13(1):38. doi:10.1186/s13584-024-00625-9.
204. Koletzko B, da Dalt L, De Guchtenaere A, et al. European Academy of Paediatrics demands protection of children's rights endangered by the ongoing Israel Gaza conflict. *Acta Paediatr*. September 2024. doi:10.1111/apa.17400.

205. Holail S, Saleh T, Xiao X, et al. Time-series satellite remote sensing reveals gradually increasing war damage in the Gaza Strip. *Natl Sci Rev*. 2024;11(9):nwae304. doi:10.1093/nsr/nwae304.
206. Saputra R, Suhardita K, Suarta IM, Aminah S, Ferdiansyah M, Ramadhani E. Prenatal nutrition and mental health: Long-term effects of hunger in Gaza and health policy implications. *Asian J Psychiatr*. 2024;101:104208. doi:10.1016/j.ajp.2024.104208.
207. Devi S. Calls for Gaza ceasefire to tackle poliovirus. *Lancet*. 2024;404(10455):837. doi:10.1016/S0140-6736(24)01776-8.
208. Alser K, Mallah SI, El-Oun YRA, et al. Trauma care supported through a global telemedicine initiative during the 2023-24 military assault on the Gaza Strip, occupied Palestinian territory: a case series. *Lancet*. 2024;404(10455):874-886. doi:10.1016/S0140-6736(24)01170-X.
209. Qureshi YA, Dawas K, Maynard ND. Excess deaths in Gaza. *Lancet Glob Health*. 2024;12(9):e1395. doi:10.1016/S2214109X(24)00267-5.
210. Zayed D, Banat M, Al-Tammemi AB. Infectious diseases within a war-torn health system: The re-emergence of polio in Gaza. *New Microbes New Infect*. 2024;62:101483. doi:10.1016/j.nmni.2024.101483.
211. Groopman J. Public Health Crisis in Gaza. *JAMA*. September 2024. doi:10.1001/jama.2024.16663.
212. Siscovick D. Public Health Crisis in Gaza. *JAMA*. September 2024. doi:10.1001/jama.2024.16660.
213. Habib AR, Ijaz N. Public Health Crisis in Gaza-Reply. *JAMA*. September 2024. doi:10.1001/jama.2024.16666.
214. Irfan B, Sultan MJ, Khawaja H, et al. Infection control in conflict zones: practical insights from recent medical missions to Gaza. *J Hosp Infect*. 2024;152:177-179. doi:10.1016/j.jhin.2024.06.014.
215. Burki T. Polio vaccination campaign in Gaza. *Lancet Infect Dis*. 2024;24(10):e623-e624. doi:10.1016/S1473-3099(24)00612-1.
216. Jaffe E, Dadon Z, Alpert EA. Prehospital Care Under Fire: Strategies for Evacuating Victims from the Mega Terrorist Attack in Israel on October 7, 2023. *Prehosp Disaster Med*. September 2024:1-4. doi:10.1017/S1049023X24000438.
217. Mohammed F, Elgailani USA, Ibrahim Ali SY, Mohamed RFA, Su Yin ET, Bravo-Vasquez ML. Defending the right to health in Gaza: a call to action by health workers. *Confl Health*. 2024;18(1):57. doi:10.1186/s13031-024-00613-5.
218. Horino M, Zaqqout R, Habash R, et al. Food insecurity, dietary inadequacy, and malnutrition in the Gaza Strip: a cross-sectional nutritional assessment of refugee children entering the first grade of UNRWA schools and their households before the conflict of 2023-24. *Lancet Glob Health*. September 2024:S2214-109X(24)00320-6. doi:10.1016/S2214-109X(24)00320-6.
219. Nasreddine L, Jomaa L. Safeguarding the SDG promise to end hunger and leave no one behind: the plight of children in the Gaza Strip. *Lancet Glob Health*. September 2024:S2214-109X(24)00367-X. doi:10.1016/S2214-109X(24)00367-X.
220. Mallapaty S. Gaza: Why is it so hard to establish the death toll? *Nature*. 2024;634(8032):18-19. doi:10.1038/d41586-024-02508-0.
221. Kienzler H, Daniel G, Hammoudeh W, Nashashibi R, Abu-Jamei Y, Giacaman R. Unbearable suffering: mental health consequences of the October 2023 Israeli military assault on the Gaza Strip. *BMJ Glob Health*. 2024;9(9). doi:10.1136/bmjgh-2023-014835.
